# Detection of the local adaptive and genome-wide associated loci in southeast Nigerian taro (*Colocasia esculenta* (L.) Schott) populations

**DOI:** 10.1186/s12864-023-09134-6

**Published:** 2023-01-24

**Authors:** Tilahun Wondimu Fufa, Temesgen Matiwos Menamo, Wosene Gebreselassie Abtew, Charles Okechukwu Amadi, Happiness Ogba Oselebe

**Affiliations:** 1 Department of Horticulture, Oromia Agricultural Research Institute, Addis Ababa, Ethiopia; 2 Department of Crop Production and Landscape Management, Ebonyi State University, Abakaliki, Nigeria; 3 Department of Plant Science and Horticulture, Jimma University, Jimma, Ethiopia; 4 Cocoyam Improvement Programme, National Root Crops Research Institute, Umudike, Nigeria

**Keywords:** Genome-environment association, Genome-traits association, Nigerian taro

## Abstract

**Background:**

Taro has a long history of being consumed and remains orphan and on the hand Nigeria farmers. The role of farmer-driven artificial selection is not negligible to fit landraces to a particular ecological condition. Limited study has been conducted on genome-wide association and no study has been conducted on genome-environment association for clinal adaptation for taro. Therefore, the objective of this study was to detect loci that are associated with environmental variables and phenotype traits and forward input to breeders. The study used 92 geographical referred taro landraces collected from Southeast (SE) Nigeria.

**Results:**

The result indicates that SE Nigerian taro has untapped phenotype and genetic variability with low admixture. Redundancy analysis indicated that collinear explained SNP variation more than single climatic variable. Overall, the results indicated that no single method exclusively was able to capture population confounding effects better than the others for all six traits. Nevertheless, based on overall model performance, Blink seemed to provide slight advantage over other models and was selected for all subsequent assessment of genome-environment association (GEA) and genome-wide association study (GWAS) models. Genome scan and GEA identified local adapted loci and co-located genes. A total of nine SNP markers associated with environmental variables. Some of the SNP markers (such as S_101024366) co-located with genes which previously reported for climatic adaptation such as *astringency*, *diaminopimelate decarboxylase* and *MYB* transcription factor. Genome-wide association also identified 45, 40 and 34 significant SNP markers associated with studied traits in combined, year 1 and year 2 data sets, respectively. Out of these, five SNP markers (S1_18891752 S3_100795476, S1_100584471 S1_100896936 and S2_10058799) were consistent in two different data sets.

**Conclusions:**

The findings from this study improve our understanding of the genetic control of adaptive and phenotypic traits in Nigerian taro. However, the study suggests further study on identification of local adaptive loci and GWAS through collection of more landraces throughout the country, and across different agro-ecologies.

**Supplementary Information:**

The online version contains supplementary material available at 10.1186/s12864-023-09134-6.

## Background

Because of worldwide environmental change, species need to adjust to the evolving climate, and this is just conceivable assuming there is adequate versatile hereditary variety at the hereditary level [1, 2]. Genome scan and genotype-environment association (GEA) techniques are utilized in the review of genetic relationship with environment. GEA is based on an alternate principle on genetic discrepancy; it accepts that adaptive loci are significantly associated with environmental variables [3]. Genome-wide scan generally depends on the assumption that the loci are considered outliers when stronger differentiation among populations and involved in adaptation [4]. Currently, there is an increasing number of literatures indicating the possibility of genome scan and GEA in detecting loci related to adaptation in cereals. Westengen et al. [5] detected adaptive loci associated with the annual precipitation and maximum temperature in African maize landrace populations. Similarly, Abebe et al. [6] identified putative adaptive loci among Ethiopian barley landraces gene pool of the farming communities. Olatoye et al. [7] also reported clinal adaptation along the West African precipitation gradient in sorghum. Similarly, GEA was also found in annual temperature and precipitation in Ethiopian sorghum landraces [8].

Taro [*Colocasia esculenta* (L.) Schott] is one of the world’s most ancient food crops, with a history of more than 2000 years of cultivation in Nigeria [9]. It is believed that taro originated in the Indo-Asian Peninsula over 50,000 years ago [10]. It arrived West Africa through the voyagers of East coast of Africa over 2000 years ago [11].

Taro is morphologically diverse with over 10,000 landraces worldwide [12] and about 10 ecotypes have been reported growing in Nigeria [13]. According to Food and Agriculture Organization of the United Nation report in 2020, Nigeria produced about 2.3 million tonnes in 0.8 million hectares with average yield of 3.98 t/ha taro. It is a highly heterozygous and clonally propagated crop with various polyploidy chromosomes: diploid (2n = 2x = 14 and 28) and triploid (2n = 3x = 42) [11, 14, 15]. Taro has a long history of being consumed for ~ 9000 years in Nigeria [16]. It is a staple food, mainly for resource-poor rural dwellers in Southeast Nigeria [13], and regularly consumed as a main component or as soup thickener [17]. However, the taro crop in Nigeria remains orphan and on farmers’ hands. This is true in most Sub-Saharan countries [18]. It is also neglected in recent advances in molecular biology appearing only in a limited number of studies utilizing next-generation transcriptome and genome sequencing [12, 19, 20].

The bulk of Nigerian taro is produced in the humid forest and derived savannah agro-ecological zones which encompass the southwest and southeast part of the country [21]. Even though the area is low in altitude, high temperature, and rainfall differences, it is much known for high taro production. Mostly farmers prefer growing taro landraces in Nigeria [22, 23]. Farmer-driven artificial selection is not negligible to fit landraces into a particular ecological condition. Limited study has been conducted on the GEA and GWAS for clinal adaptation for taro. Therefore, the objective of this study was to detect alleles that are associated with environmental variables and phenotype traits with the idea that these alleles may confer a selective advantage in Southeast Nigerian environment.

## Materials and methods

### Field experiment

The field experiment was carried out at Ebonyi State University (EBSU), Abakaliki, Nigeria in two cropping seasons (2018 and 2019). The experiment was laid out using alpha lattice design with three replications.

### Genetic materials

**Fig. 1:**
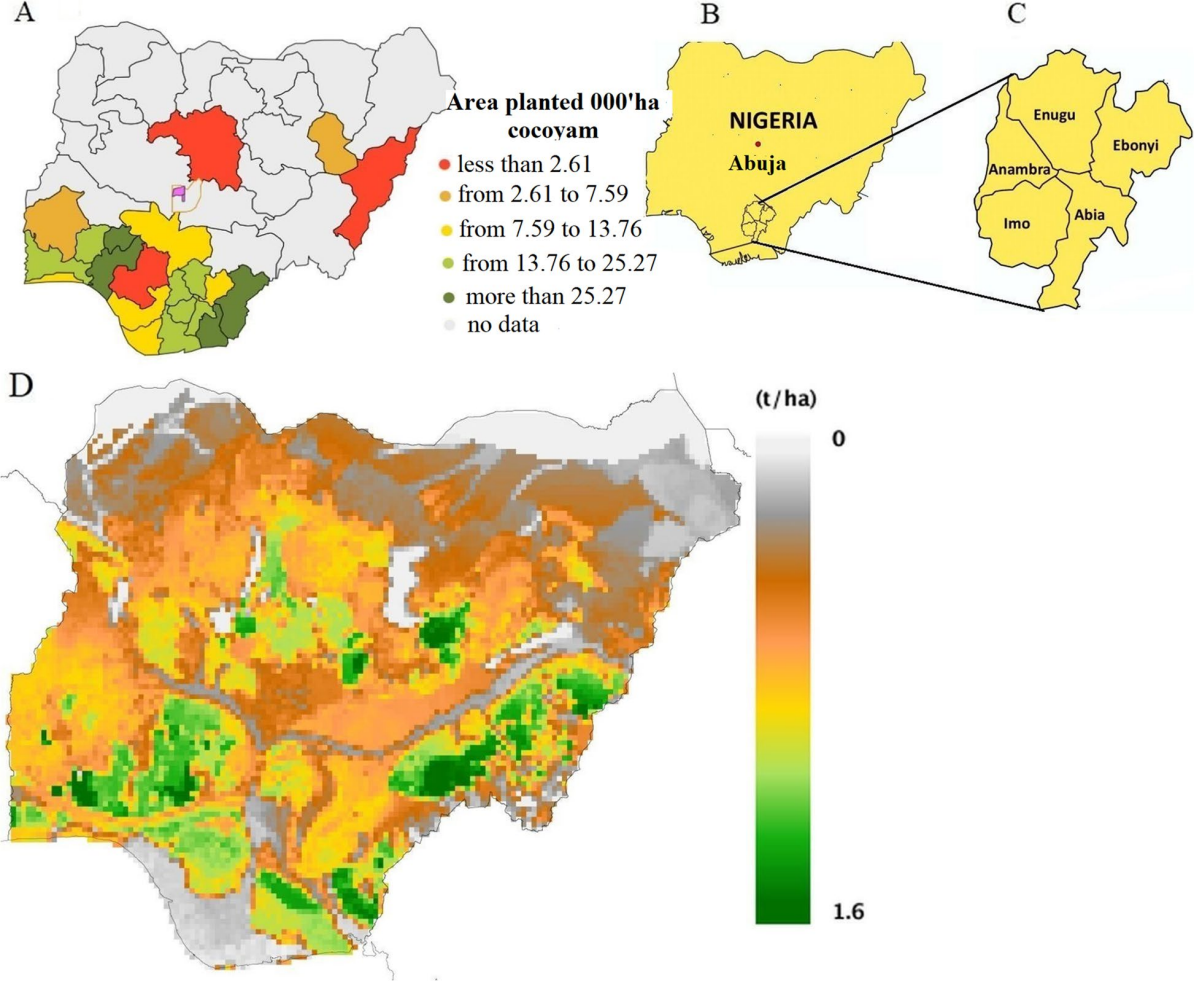
Map of Nigerian taro production status (**A**), landraces collection states (Southeast states (**B** & **C**) and total potential production (**D**)

A total of 92 diverse taro germplasm landraces were used in this study (Table S1). The genetic material was collected from Southeast states of Nigeria in 2015 (Fig. 1 B and C). The information on taro production regions (Fig. 1) and the availability genetic resource during collection season were used as criteria to systematically select representative samples from Southeast states of the country. Southeast states produce 13,760 to 25,270 ha of cocoyam i.e., including Taro (Fig. 1A). The states contain high to low potential production areas (Fig. 1D). The collection covered all taro producing areas in Southeast states i.e., low to high producer areas.

#### Total genomic DNA extraction and genotyping

The 92 taro landraces were grown at EBSU during 2018 and 2019 cropping seasons. Young taro leaf samples were collected for each landrace at 2 months old stage and dried using silica gel. The dried leaf samples were shipped to Biosciences Eastern and Central Africa (BecA-ILRI) Hub, Nairobi for genomic DNA extraction and Genotyping.

DNA extraction was done using Nucleomag Plant DNA extraction kit. Libraries were constructed using a combination of *P*st*I* and *MseI* restriction enzymes [24] and use site-specific adapters for barcode adapter ligation followed by PCR amplification. Libraries were sequenced by means of single read sequencing runs for 77 bases. Next generation sequencing was carried out using Hiseq2500. DArTseq markers scoring was attained using DArTsoft14 which is an in-house marker scoring software based on algorithms. DArTseq markers genotyping was scored as binary form presence /absence (1and 0, respectively). DArT markers were aligned to the reference genomes of Taro (Taro_V1) to identify chromosome positions. The integrated genotyping support and service (IGSS) platform uses a genotyping by Sequencing DArTseqTM technology.

#### Climatic and phenotype variables

Climate variables (average from 1960 to 1990) were extracted from WorldClim 1.4 using the *Raster* package in R [25] based on the coordinate (latitude and longitude) for each of the 92 georeferenced Nigerian landraces (Table 1) five environmental layers (30 arc sec resolution, i.e., ~ 1 km) [26]. Phenotypic variables such as corm diameter, corm length, cormel diameter, cormel length, cormel weight, dry matter, number of cormels per plant, plant height, number of leaves per plant, number of suckers per plant, petiole length, yield per hectare, and yield per plants for landraces were obtained from two cropping seasons (2018 and 2019) data from EBSU field experiments. Taro descriptor [27] was used for data collection (Table 1). The data was collected from five randomly selected plants except for yield that was taken from the whole plot and converted to per hectare.

**Table 1:** Description for phenotype data collection from 92 taro accessions

Phenotype traits	Description*
Corm diameter (cm)	It was measured from maximum circumference of corm plants using calliper.
Corm length (cm)	It was measured from the distal end of the corm to the proximal end where the outer leaf petiole is attached to the corm using calliper
Cormel diameter (cm)	It was measured at the maximum circumference of the cormel using calliper
Cormel length (cm)	It was measured from the distal end of the cormel to the proximal using calliper
Cormel weight (g)	It was measured the weight using sensitive balance
Days to maturity	It was counted days from planting to harvesting of the yield.
Number of cormels per plant	It was counted the number of cormels per plant at harvesting
Plant height (cm)	It was measured from collar region to the attachment point between the leaf petiole and the lamina of the tallest leaf by meter
Number of leaves per plant	It was counted all leaves starting emergence to physiological maturity
Number of suckers per plant	It was counted all suckers per plant at maturity stage
Petiole length (cm)	It was measured from based of the petiole to the attachment point of the tallest leaf
Yield per hectare (t/ha)	Total (Corm and cormel) yield was measured from plot based and converted to t/ha
Yield per plants (kg)	It was measured all corm and cornels using sensitive balance

#### Morphological data analysis

The best linear unbiased estimate (BLUEs) was used to estimate trait values of the 2 years (2018 and 2019) combined and individual year for each landrace. The BLUE model using *lmer* function in *LME4* package of R [28] was as follows:

*y*_*i*_ = *μ* + *Landrace*_*i*_ + *Block*_*j*_ + *Season*_*k*_ + (*Landrace x Block*)_*ij*_ + (*Landrace x Season*)_*ik*_ + *ε*_*ijk*_. Where μ is the mean, Landrace_i_ is the genotype effect of the i^th^ landrace, Block_j_ is the effect of the j^th^ Block, Season_k_ is the effect of the k^th^ year, (Landrace x Block)_ij_ is the Landrace-Block interaction effect, (Landrace x Season)_ik_ is the landrace-season interaction effect, ε_ijk_ is the error of the j^th^ block in the k^th^ year. Landrace was considered as fixed effect whereas all remaining items are considered as random effects for estimation of heritability and BLUEs. The coefficient of variation (environment, genotype and phenotype), heritability and genetic advance were estimated using *variability* R package [29].

#### Population structure and linkage disequilibrium analyses

Pairwise linkage disequilibrium (LD) (r^2^) was analysised using TASSEL 5 software [30]. The LD decay plot was constructed using Remington et al. [31] procedure in R software [32]. The population structure investigation was done using *LEA* (Landscape and Ecological Association Studies) version 1.8.1 in R [33, 34].

#### Redundancy analysis

Redundancy analysis (RDA) was carried-out using R *vegan* package and *varpart* function [35]. A multivariate model was fitted using 9442 filtered SNP markers as response variable. Annual mean temperature and precipitation as climatic variables; latitude and longitude as geographical variables (“space”) and altitude were fitted as predictor terms. The “space” term is used to account for isolation-by-distance [36]. The altitude variable was used based on the altitude of the collection area, as identified during sample collection using GPSMAP 64x handheld navigator. The default function of the package was used to test the significance of the proportion of variation explained by climate collinear with space in the germplasm. Finally, 1000 permuted data set was used to compare the distribution in variation explained. In each stage of the permutation, genotype were randomized and RDA regression fitted and repeated 1000 times.

### Detect local adaptation loci

Genome scan was performed using *pcadapt* R package for detecting local adapted loci [37]. This was first done by using a PCA with a number of groups (K) equal to the number of subpopulations investigated to define the optimal value for K. Benjamini & Hochberg Procedure [38] was used to determine false discovery rate (FDR) of *p* values distribution at 0.05. Finally, a list of outlier loci obtained that were candidates for selected loci.

#### Genome–environment association studies

Genome–environment association studies (GEA) were analysed using ten environmental variables. These are annual mean temperature, mean temperature of driest quarter, mean temperature of wettest quarter, mean temperature of warmest quarter, mean temperature of coldest quarter, annual mean precipitation, precipitation of wettest quarter, precipitation of driest quarter, precipitation of warmest quarter and precipitation of coldest quarter. The variable data were averaged from 30 years (1960 to 1990). GEA was performed using *GAPIT3* R package [39].

#### Genome-wide association studies

Genome-wide association studies (GWAS) were performed using BLUEs in traits values of 2 years both combined and individual. Population structure and genetic relationships among accessions were used to minimize false-positive associations. Population structure represented by the PC was estimated with the *GAPIT3* package [39].

### Setting significant threshold *P* values and model validation for GWAS and GEA

We set the suggestive significant threshold using a multiple testing correction developed by Li and Ji ($\alpha \ast =1-{\left(1-{\alpha}_F\right)}^{ {1}\!\left/ \! { Meff }\right.}$) to identify significant loci underlying variables [40]. Whereas, α* = suggestive significant threshold, α_F_ = alpha value (*P* = 0.05) and Meff = effective number of markers. Meff and α* were estimated using *poolr* R package [41].

Fitness of different GWAS and GEA models for all variables was evaluated using Quantile-Quantile (Q-Q) plots of the observed versus expected –log10(p) values which should follow a uniform distribution under the null hypothesis and genomic inflation factor (λ). In order to compare how well the models adjusted for systematic effects, the genomic inflation factor (λ) for all methods was calculated in R software as follows:

$$\uplambda =\frac{M}{E}$$

Where λ is the genomic inflation factor and M is median of the resulting chi-squared test statistics and E is the expected median of chi-squared distribution [42].

## Results

### Phenotype traits and environmental variability

Analysis of variance indicated the presence of highly significant difference (*P < 0.001*) in all morphological traits studied among the landraces. Number of leaves per plant varied from 7.40 to 12.40; number of suckers per plant varied from 2.50 to 14.40; petiole length varied from 16.67 to 6.00 cm; corm diameter varied from 2.62 to 11.06 cm (Table 2). The corm yields also varied from 0.05 to 1.16 kg per plant and 1.40 to 18.03 t/ha. The genetic coefficient of variation was high in all traits compared to environment coefficient of variation except cormel weight, days to maturity, number of cormels per plant, number of leaves per plant and petiole length. The heritability estimate varied between 0.24 (number of cormels per plant) to 0.75 (corm diameter). The genetic advance as a percentage of mean also varied from 6.10 (days to maturity) to 63.91 (yield per hectare). The climatic variables showed variation among the collected areas. The extracted climatic variables and mean BLUEs phenotype traits are described detail in Table S2 and S3, respectively.

**Table 2:** Mean, range, genetic variability among 92 Nigerian taro landraces

Trait	Mean	Min	Max	ECV	GCV	PCV	CV	*H*^*2*^	GA	Sing.
COD	6.74	2.62	11.06	11.43	19.71	22.79	10.00	0.75	35.13	< 0.001
COL	6.70	2.26	9.36	11.55	16.00	19.73	11.59	0.66	26.72	< 0.001
CRD	3.71	2.45	5.95	8.64	12.60	15.28	8.53	0.68	21.40	< 0.001
CRL	5.78	3.40	8.38	11.62	12.69	17.21	11.46	0.54	19.28	< 0.001
CRW	39.71	16.21	84.30	21.82	19.98	29.59	21.25	0.46	27.80	< 0.001
DM	197.19	178.00	213.00	4.31	4.23	6.05	4.14	0.49	6.10	< 0.001
NCR	11.36	0.00	45.00	45.30	25.76	52.11	40.63	0.24	26.23	< 0.001
NLPP	9.63	7.40	12.40	7.89	7.86	11.13	7.40	0.50	9.28	< 0.001
NSPP	7.75	1.00	14.40	23.40	26.19	35.12	23.20	0.55	40.22	< 0.001
PH	75.28	35.00	110.00	13.13	15.38	20.22	12.63	0.58	24.09	< 0.001
PL	31.82	15.65	69.00	16.82	15.86	23.12	16.30	0.47	22.41	< 0.001
YPH	10.08	1.40	18.03	24.04	37.00	44.12	23.60	0.70	63.91	< 0.001
YPP	0.68	0.05	1.16	23.70	28.14	36.78	22.50	0.58	44.35	< 0.001

*COD* Corm diameter (cm), *COL* Corm length (cm), *CRD* cormel diameter (cm), *CRL* Cornel length (cm), *CRW* Cormel weight (g), *DM* Days to maturity, *NCR* Number of cormels per plant, *PH* Plant height (cm), *NLPP* Number of leaves per plant, *NSPP* Number of suckers per plant, *PL* Petiole length (cm), *YPH (t/ha)* Yield per hectare, and *YPP* Yield per plants (kg/plant), *SD* Standard deviation, *Min* Minimum, *Max* Maximum, *ECV (%)* Environmental Coefficient of Variance, *GCV (%)* Genotypic Coefficient of Variance, *PCV (%)* Phenotypic Coefficient of Variance, *CV (%)* Coefficient of variation, *H*^*2*^ broad-sense heritability, *GA(%)* Genetic advance as percentage of mean, *sign.* significance *p*-values

### SNP markers and linkage disequilibrium (LD) decay analyses

**Fig. 2:**
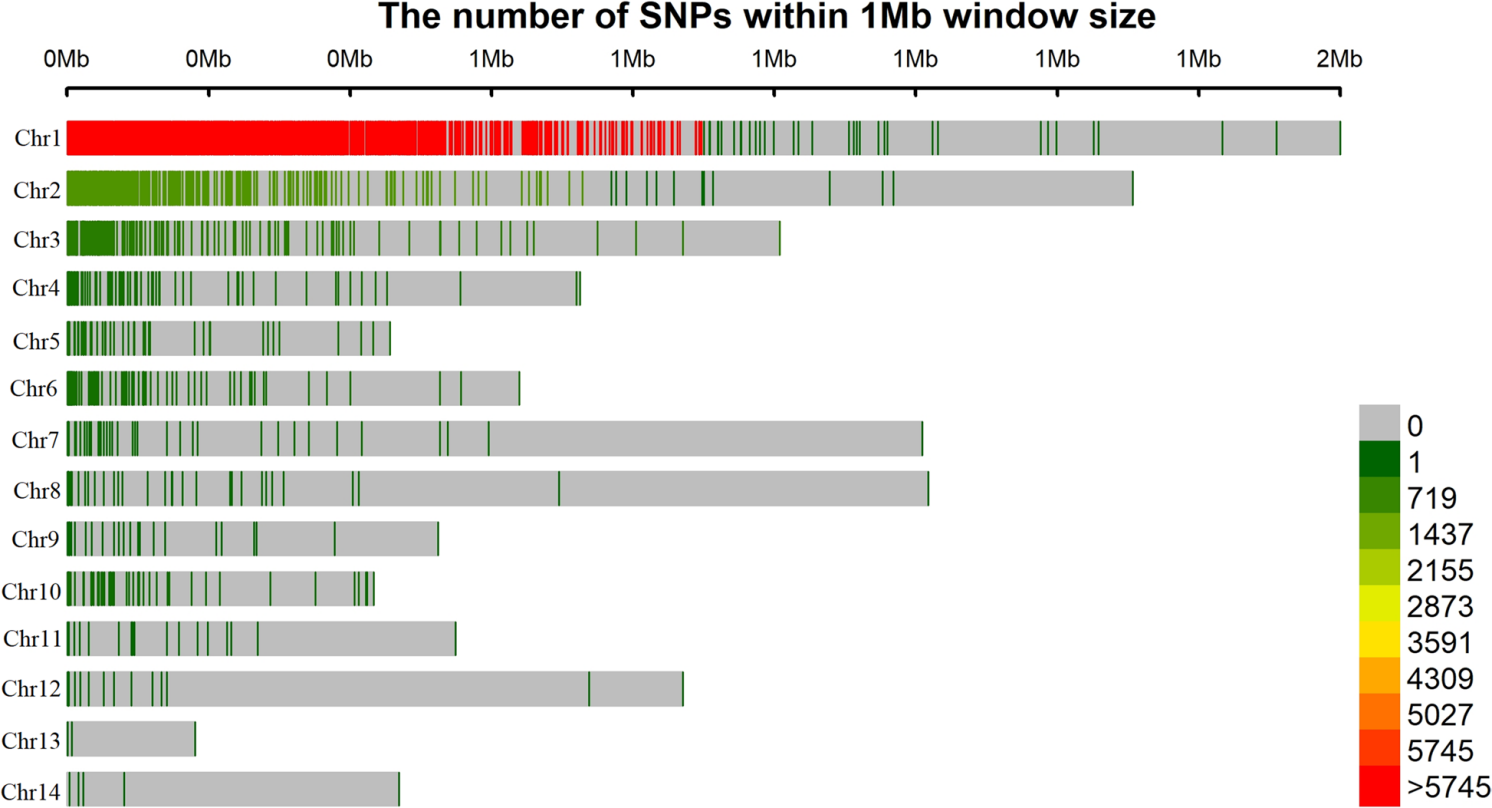
Distribution and density of filtered SNP markers across 14 chromosomes. The horizontal axis displays the chromosome length. The number of SNPs in a given region is indicated at the bottom right side

Ninety-two Nigerian germplasm landraces and 32,327 SNP markers were identified in the study. Following exclusion of markers with > 25% missing values, non-chromosome positions, redundant markers and MAF < 0.05, a subset of 9442 SNP markers were identified and missing values inferred using the Beagle 5.0 software [43]. The density of markers is unevenly distributed across the chromosomes (Fig. 2). Large number of SNP markers were located on chromosome Chr1. The detail of the marker description was presented in Fufa et al. [44].

**Fig. 3:**
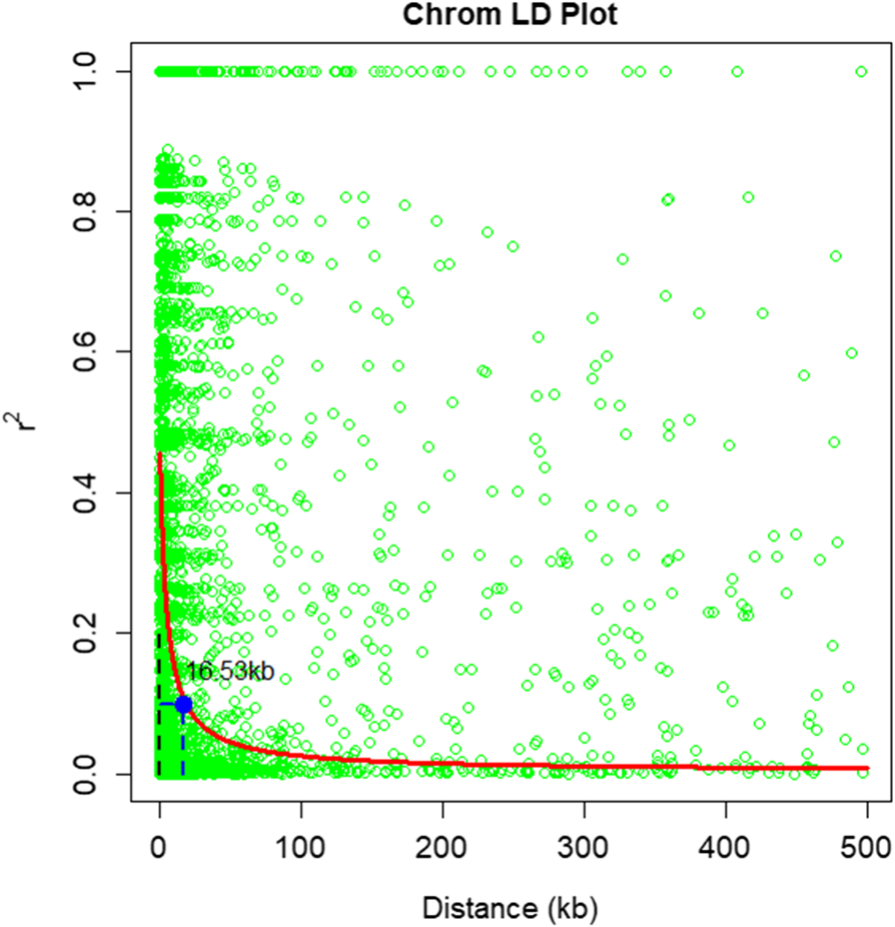
Linkage disequilibrium (LD, *r*^*2*^) decay plot of 9442 marker pairs as a function of kilo base pair (kb) for the 92 taro landraces used in this study

Pairwise LD, estimated using the squared allele frequency correlations (*r*^*2*^), decayed rapidly at *r*^*2*^ *= 0.1* with kilobase pair (Fig. 3). Approximately 2.67% of these comparisons had a significant LD value, and the mean *r*^*2*^ was 0.388. The average LD decay distance was about 16.53 kb for locus pairs with *r*^*2*^ *= 0.1* at the whole genome level.

### Population structure

**Fig. 4:**
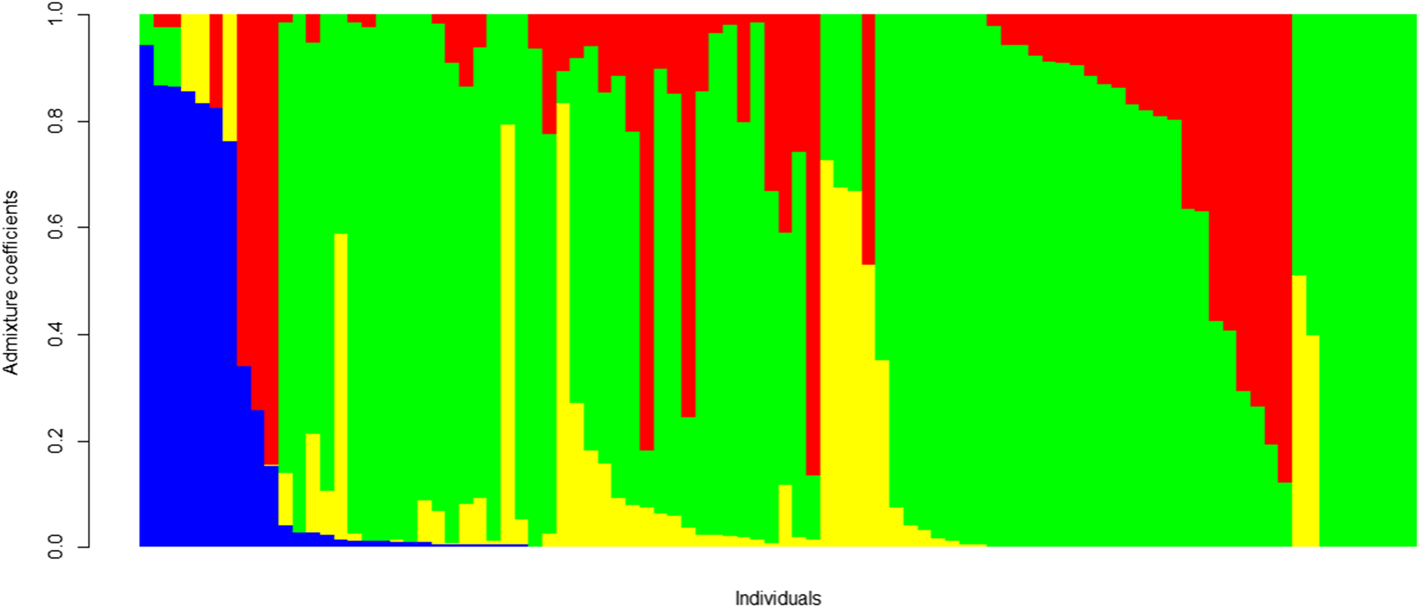
Population structure for k = 4 in Nigerian taro landraces using SNP markers. Each vertical line represents one accession, and the color composition displays the probability of belonging to each of the 4 subpopulations

Population structure analysis among 92 taro landraces with a set of 9442 SNP markers suggested optimum K value of four, representing the landraces into four major subpopulations (Fig. S1). First subpopulation comprised seven landraces and the second subpopulation comprised eight. The third and fourth subpopulations comprised 65 and 12 landraces, respectively. The structure results mainly supported the population structure analysis with 93.5% of the genotypes being assigned to one of the four subpopulations with a higher than 0.60 ancestry membership coefficient (Fig. 4). Hence, only 6.5% were identified as admixture landraces.

#### Redundancy analysis

**Fig. 5:**
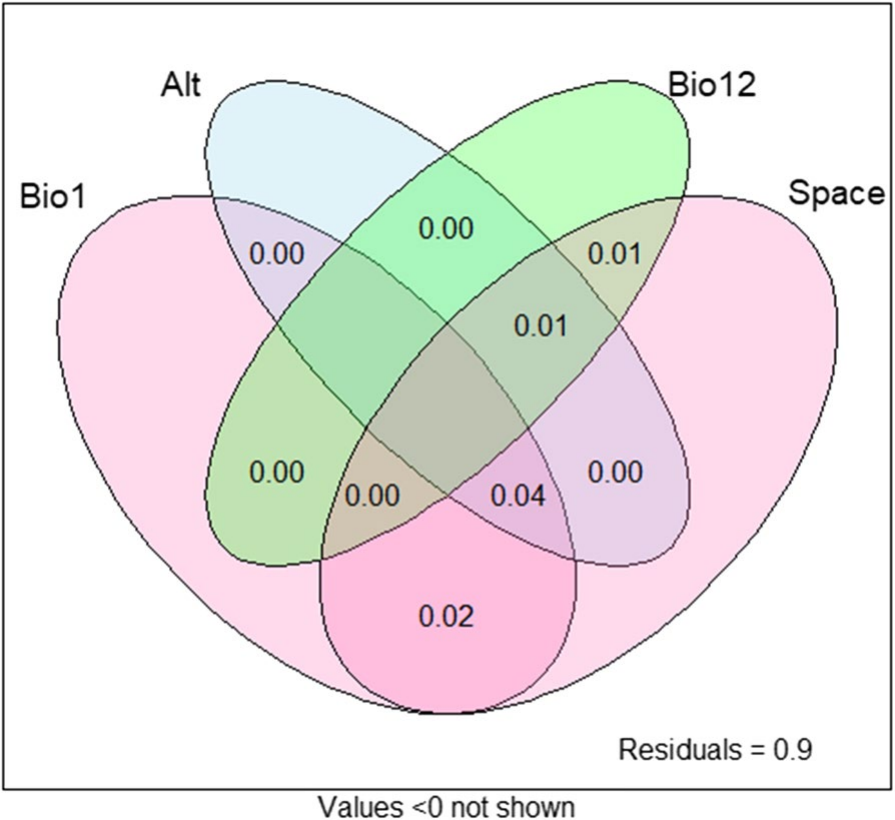
Redundancy analysis of SNP variation explained by climatic and spatial variables. A) Annual temperature (BIO1), Altitude (Alt), annual precipitation (BIO12) and space (latitude and longitude); and B) climate variables (precipitation and temperature) and space (latitude and longitude)

Redundancy analysis was performed to estimate the proportion of SNP variation explained by agro-climate and geographical locations (Fig. 5). This analysis indicated none of variable alone contributed for SNP variation rather than in collinear. Hence, the larger (4%) variation was explained by the collinearity of annual temperature, altitude and space (geographical location). Annual temperature, annual rainfall and space together explained the SNP variation only 1%.

### Suggestive *p*-value and model validation using GWAS results

According to Li and Ji (2005), a total of 137 effective SNP markers (Meff) was identified out of 9442 SNP markers. Using Meff, the suggestive threshold *P*-value was estimated *3.74e-4* (−log10(p) = ~ 3). We demonstrated here the effectiveness of different models for performing Genome-environment association (GEA) and genome-wide association (GWAS) in taro. Performance of GEA models was evaluated using Q-Q plots of the expected versus observed –log10(*p*) values and genomic control inflation factors (λGC) achieved for each ‘variable x model’ combination. None of the models was suitable for all environmental variables and agro-morphological traits based on genomic control inflation factors (Table S4, Fig. S2 and S3). However, GLM and FarmCPU models were the least compared to others. These two models are only best for days to maturity than other models. In general, Blink was better in seven environmental variables and three phenotypic traits. MLM was better for four environmental variables and six phenotypic traits.

### Genome scan and environment association loci

A genome scan was performed using *pcadapt* R package to detect the outliers SNP markers. For further identification of the outliers, GEA was performed (Fig. S4). Only true marker-environment associations i.e. (a) from models where *p*-value inflation was close to the expected normal value (λGC ~ 1) and (b) which passed the set threshold are included for reporting GWAS results. Pcadapt analysis detected 2355 outlier SNPs with the threshold values alpha < 0.1 using Benjamini & Hochberg Procedure [38]. Out of 2355 outliers, GEA identified only nine SNP markers associated with environmental variables (Table 3 and Fig. S2). All Associated SNP markers were identified as outliers in genome scan except *S_100830796* and *S_100913593* markers. Specifically, *S_101024366* marker was significantly associated with all environmental variables except precipitation of warmest quarter. *S_100991964* SNP marker significantly associated with all precipitation variables except precipitation of warmest quarter. Indeed, no genome association was detected with precipitation of warmest quarter. *S_100830796* SNP marker associated with all temperature variables such as annual mean temperature, mean temperature of wettest quarter, mean temperature of driest quarter, mean temperature of warmest quarter, and mean temperature of coldest quarter. *S_100583021* SNP marker significantly associated with mean temperature of annual mean temperature and warmest quarter.

**Table 3:** List of SNP markers identified by genome-environment association with their effects

Environment variables	SNP	Chr	Pos (bp)	*P* value	Effect	R^2^
BIO1	S1_101024366	1	12,021	2.5E-05	−0.86	0.16
	S1_100830796	1	392,983	2.2E-04	0.71	0.13
	S1_100583021	1	899	5.4E-04	0.74	0.11
	S2_18902671	2	8305	3.9E-04	0.21	0.12
	S1_100583021	1	899	5.4E-04	0.74	0.11
BIO8	S1_101024366	1	12,021	1.0E-04	−0.87	0.17
	S1_100830796	1	392,983	6.5E-04	0.69	0.12
	S2_18902671	2	8305	7.2E-04	0.20	0.12
BIO9	S1_101024366	1	12,021	1.1E-04	−0.95	0.16
	S1_100830796	1	392,983	7.0E-04	0.76	0.12
	S2_18902671	2	8305	7.5E-04	0.23	0.12
BIO10	S1_100583021	1	899	3.7E-04	0.76	0.14
	S1_101024366	1	12,021	4.0E-04	−0.73	0.14
	S1_100830796	1	392,983	4.3E-04	0.68	0.14
	S2_100587991	2	50,903	5.3E-04	0.86	0.13
BIO11	S1_101024366	1	12,021	1.0E-04	−0.87	0.17
	S1_100830796	1	392,983	6.5E-04	0.69	0.12
	S2_18902671	2	8305	7.2E-04	0.20	0.12
BIO12	S1_100991964	1	6698	3.0E-05	− 231.89	0.15
	S1_101024366	1	12,021	8.3E-05	−200.50	0.14
	S1_100379892	1	388,151	2.4E-04	− 155.49	0.12
	S5_18911928	5	816	2.9E-04	−75.08	0.12
	S2_100913593	2	167,750	5.0E-04	128.23	0.11
	S2_18902671	2	8305	5.0E-04	22.30	0.11
BIO16	S1_100991964	1	6698	2.2E-05	−95.56	0.16
	S1_101024366	1	12,021	3.1E-05	− 85.78	0.15
	S1_100379892	1	388,151	2.3E-04	64.01	0.12
	S5_18911928	5	816	5.4E-04	−29.20	0.11
	S2_18902671	2	8305	5.8E-04	10.33	0.11
BIO17	S1_100991964	1	6698	7.3E-05	−15.20	0.14
	S5_18911928	5	816	2.3E-04	−5.23	0.12
	S1_100379892	1	388,151	3.1E-04	10.60	0.11
	S2_100913593	2	167,750	3.4E-04	9.06	0.11
	S1_101024366	1	12,021	4.2E-04	−12.43	0.11
BIO18	S1_100991964	1	6698	7.9E-04	−46.45	0.13
BIO19	S1_100991964	1	6698	2.2E-05	−95.56	0.16
	S1_101024366	1	12,021	3.1E-05	−85.78	0.15
	S1_100379892	1	388,151	2.3E-04	64.01	0.12
	S5_18911928	5	816	5.4E-04	−29.20	0.11
	S2_18902671	2	8305	5.8E-04	10.33	0.11

*BIO1* Annual mean temperature, *BIO8* Mean temperature of wettest quarter, *BIO9* Mean temperature of driest quarter, *BIO10* Mean temperature of warmest quarter, *BIO11* Mean temperature of coldest quarter, *BIO12* Annual precipitation, *BIO16* Precipitation of wettest quarter, *BIO17* Precipitation of driest quarter, *BIO18* Precipitation of warmest quarter and *BIO19* Precipitation of coldest quarter, *SNP* Single nucleotide polymorphism, *Chr* Chromosome, *Pos* Position (bp = base pair), *R*^*2*^ Variance explained by the marker

### Genome-wide association

Based on the suggestive *p*-value (*P < 9.05E-04*) threshold, significant SNP markers were identified to be associated with studied traits on chromosomes 1, 2, 3, 4, 5, 9 and 10 based on three data set (two separate data and combined one) (Table 4 and Fig. S5A-C). Even though none of the markers were identified across the data sets, some markers were identified at least in two data sets. For example, S1_18891752 SNP marker which is associated with cornel weight was identified in both combined and Year 1 (2018) data sets. Other markers such as S3_100795476 associated with dry matter were identified in combined and Year 2 data sets; S1_100584471 associated with number leaves per plant in combined and year 1 data sets; S1_100896936 associated with yield per hectare in combined and year 1 data sets; and S2_100587991 associated with yield per hectare in combined and year 2 data sets.

**Table 4:** List of SNP markers identified by genome-wide association using combined and separate data (2018 & 2019). SNPs in bold are those significantly associated at least in two data sets

	Combined data				Year 1 (2018) data				Year 2 (2019) data			
Traits	SNP	Chr	Pos (bp)	*P* value	SNP	Chr	Pos (bp)	*P* value	SNP	Chr	Pos (bp)	*P* value
COD	S3_101063428	3	50,039	4.46E-04	S1_100689072	1	202,417	6.46E-05	S1_100693199	1	28,956	8.28E-04
					S1_100997735	1	54,446	8.13E-05				
					S2_18893366	2	2096	8.54E-05				
					S2_100944099	2	275,709	3.72E-05				
					S4_18895871	4	22,187	4.58E-05				
					S2_100756057	2	284,159	5.64E-05				
					S1_100842346	1	109,989	5.71E-05				
					S1_100802428	1	228,448	8.09E-05				
					S10_18892924	10	1320	8.42E-05				
COL	S1_100927695	1	171,377	0.0005	S1_100374249	1	14,226	1.32E-04	S1_100693199	1	28,956	7.28E-04
	S1_100374249	1	14,226	0.000769	S2_100809767	2	97,399	2.78E-04				
	S1_100934305	1	53	0.000839	S1_100927695	1	171,377	3.39E-04				
					S7_18912893	7	134	3.47E04				
					S6_101039431	6	17,094	4.23E-4				
					S12_100811923	12	15,826	6.91E-04				
CRD	S2_100843507	2	1849	8.89E-05	S3_100998987	3	7027	0.000473	S1_100693199	1	28,956	6.28E-04
	S3_100998987	3	7027	0.000337	S1_100574592	1	6750	0.000836				
	S1_100574591	1	6750	0.000367	S1_100582688	1	39,446	0.000864				
	S3_101010746	3	381	0.000399								
	S1_100582688	1	39,446	0.000417								
CRL	S1_18896887	1	116,872	0.000591					S1_100892740	1	27,909	0.000442
	S1_100681096	1	10,631	0.000667	NA	NA	NA	NA	S6_100839526	6	171,520	0.000626
	S1_100892740	1	27,909	0.000792					S7_100839525	7	703	0.000626
CRW	**S1_18891752**	1	199,716	0.000589	**S1_18891752**	1	199,716	3.31E-08	S4_100974332	4	10,835	0.000464
					S2_18891794	2	93,043	1.65E-05	S1_100678415	1	246,142	0.00089
DM	S1_100579053	1	13,403	0.000121	S1_100677594	1	117,831	0.000249	S2_100587991	2	50,903	0.000175
	S1_18896636	1	361,107	0.000184	S1_18905350	1	150,792	0.000896	**S3_100795476**	3	325,970	0.000899
	**S3_100795476**	3	325,970	0.000188								
	S1_100378035	1	58,608	0.000396								
	S1_100678255	1	918,125	0.000805								
NCR	S3_101024887	3	232	0.000812	S1_18896897	1	123,749	0.000836	S1_100680602	1	4856	3.07E-05
									S1_18878040	1	658,024	3.12E-05
									S1_100755696	1	49,669	0.000119
									S1_18908588	1	64,068	0.000152
									S5_100689668	5	333,890	0.000262
									S1_100375214	1	97,399	0.000286
									S10_100964255	10	606	0.000318
									S1_100990433	1	481,249	0.000458
									S1_100918481	1	53,052	0.000478
									S1_18892187	1	18,934	0.000502
									S1_100897692	1	781,657	0.000667
NLPP	**S1_100584471**	1	57,350	4.53E-05	**S1_100584471**	1	57,350	7.48E-05	S1_100693199	1	28,956	0.001452
	S9_100959651	9	9359	0.000294	S2_100685734	2	32,330	0.000512				
PH	S1_100839334	1	189,378	0.000108	S1_101059919	1	34,704	0.000363	S1_100836468	1	297,222	0.000134
	S1_18895647	1	66,399	0.00025	S1_100379454	1	379,757	0.000728	S2_100946429	2	325,580	0.00018
	S1_100579053	1	13,403	0.000386					S3_101024887	3	232	0.000465
	S1_100798136	1	34,573	0.000729					S2_100839596	2	17,956	0.000706
	S2_100801959	2	1457	0.000751								
PL	S1_100765786	1	34,841	0.00024	S2_100688934	2	113,866	1.15E-07	S1_100747947	1	230,442	5.17E-05
	S2_100874072	2	215,874	0.000242	S1_100381240	1	479,002	0.000264	S2_100892757	2	29,173	6.05E-05
	S1_100898140	1	43,180	0.000375	S1_100681342	1	3737	0.000284	S1_100836468	1	297,222	0.000104
	S10_101005079	10	377,504	0.000508	S1_100917554	1	419,632	0.000552	S2_100946429	2	325,580	0.000108
					S3_100944639	3	1101	0.000767	S1_100683489	1	91,412	0.000195
					S3_100982330	3	267,759	0.000862	S2_101010732	2	776	0.00061
YPH	S1_100379475	1	36,727	0.000636	S2_100944099	2	275,709	0.000411	S1_100592663	1	348,550	0.000211
	**S1_100896936**	1	81,628	0.000139	S1_100381260	1	156,721	0.000461	S3_100379409	3	984	0.000343
	S1_100934187	1	17,817	0.000552	S1_18896636	1	361,107	0.000669	**S2_100587991**	2	50,903	0.000553
	**S2_100587991**	2	50,903	1.56E-05	**S1_100896936**	1	81,628	0.000833	S1_18895526	1	147,250	0.000603
	S2_100688888	2	209,908	0.000495	S1_100584829	1	7043	0.000837	S5_100869929	5	102,007	0.000604
	S2_18913028	2	1,313,949	0.000723								
	S5_101068528	5	274	0.000853								
YPP	S1_100753561	1	59,137	0.000331	S1_100974034	1	2747	0.000356	S2_100587991	2	50,903	8.67E-08
					S1_100586420	1	102,751	0.000484	S2_100754258	2	757	0.000236
					S1_100802848	1	184,891	0.000883	S2_100690401	2	8539	0.000643
									S4_18904835	4	294,901	0.000941

*COD* Corm diameter (cm), *COL* Corm length (cm), *CRD* Cornel diameter 9 cm), *CRL* Cornel length (cm), *CRW* Cornel weight (g), *DM* Dry matter, *NCR* Number of cormels per plant, *PH* Plant height (cm), *NLPP* Number of leaves per plant, *NSPP* Number of suckers per plant, *PL* Petiole length (cm), *YPH (t/ha)* Yield per hectare, and *YPP* Yield per plants (kg/plant)

## Discussion

### Significance of the study for taro improvement

Farmers and breeders have focused on selecting crops with desirable phenotypes for several years [45] which leads to loss of genetic and phenotypic variation. This is the major cause for genetic bottlenecks especially when stress occurs [46]. For example, Markwei et al. [47] reported the loss of cocoyam cultivar *amankani kyirepe* and that others such as *Amankani fita* and *amankani Serwaa* face the risk of being lost. Hence, evaluation of genetic diversity and genome association study is an important step for further genetic conservation and breeding program of the crop.

Taro has a large genome estimated to be 4.08 Gbp [48]. However, currently, taro genome of only 2.2 Gbp (chromosome based) and 0.27 Gbp of unknown region is available in NCBI database submitted by Jiangsu Academy of Agricultural Sciences [49]. This is promising progress to improve our understanding of taro genetics but still needs further sequencing to a high-quality reference genome. That might have led to uneven distribution of the SNPs across the chromosomes in this study. The size of the sequenced reference genome also varied 212.14 Mbp (Chromosome 1) to 102.22 Mbp (Chromosome 12). This may also be another cause for the uneven distribution of the SNP markers across the chromosomes.

### Southeast Nigerian taro has untapped phenotypic variability

Significant variability was observed among the landraces in all studied morphological traits. The phenotypic variation among landraces was also high which more desirable for selection. Specifically, higher the genetic variation than environment variation among landraces is an indication of the potential for selection of the given trait. Corm diameter, corm length, cormel diameter, cormel length, number of suckers per plant, plant height, yield per hectare, and yield per plant traits had more genetic coefficient of variation than environmental variation. These traits might be used for clonal selection for further improvement of taro landraces. Similarly, Mukherjee et al. [50] reported that high genotypic coefficient of variability (GCV) values for weight of cormels per plant and number of cormels per plant. The trait heritability varied from medium to high except in number of cormels per plant trait. Both heritability and genetic advance were high for corm diameter, and yield per hectare. Such high heritability followed by high genetic advance indicates that clonal selection may be effective for the improvement of such characters. The phenotypic coefficient of variability (PCV) was generally higher than the GCV for all the studied traits but the differences were quite small except for the number of cormels per plant. This suggests that environmental effects constitute a less portion of the total phenotypic variation in the traits [51].

### Collinear explained SNP variation more than single climatic variable

Although RDA and LFMM are efficient methods to identify candidate SNPs associated with variability in environmental conditions [52, 53], no significant relationship was detected between any of the SNPs and climatic variables (the temperature and precipitation) alone. In total, only 10% variation of the SNP explained by collinear of altitude, annual temperature, annual precipitation, and space among 92 Nigerian taro landraces. The maximum SNPs variation (4%) is explained by the collinear of annual temperature, altitude, and space. This suggests that collinear climatic variables are more important than single climatic variables in shaping variation for taro clinal adaptation. A considerable percentage of the variance was not explained by either geographic location or climatic variables, implying that other factors such as human activities or human habitation may be important. According to recent studies, sorghum genetic structure has also been shaped via seed sharing and ethnolinguistic grouping [8, 54]. Markwei et al. [47] also reported that the development of human selection based on people’s interests and their cultural communication habits has great impact on taro diversity and distribution in China. Taro seeds that are exchanged among farmers and grown often harbour a unique genetic diversity in landraces [55].

### Southeast Nigerian taro has low admixture

The success of plant breeding is associated with accessing landraces and wild relatives of crop species for new sources of variation [56]. Hence, knowledge about the genetic diversity and the population structure of landraces is needed to access the reservoir of favourable alleles within landrace or wild germplasm. The collection (92 taro landraces) was grouped into four subpopulations with low admixture (6.5%) among the individuals in the collection. The low admixture observed is likely due to low gene flow among subpopulations or individuals in the subpopulation. This indicates the introduction of new genetic lineages into a population is low. Different studies reported that taro is not native to Africa and it reached through human migration with a single clone introduction from a single point of origin, then the accumulation of mutations leading to different multi-locus genotypes during the dissemination process [10, 11]. This may lead to loss of genetic resources due to outbreaks (such as new pests and diseases or climatic changes). Recently, loss of genetic resources started with the outbreaks of taro leaf blight disease in west Africa including Nigeria [57]. Hence, taro breeding through hybridization is important in Nigeria. However, taro is a clonally propagated crop with different polyploidy levels 2n = 2x = 14, 28, 42 [58, 59]. One, the challenge of performing cross-pollination due to the infrequent flowering habit such as rarely flowers and its flower anatomy discourages natural pollination when it does. However, Wilson and Cable [60] reported that the application of gibberellic acid-induced flower formation in taro increases the possibility of producing new taro varieties or hybrids. Another option is the introduction of the germplasm from centre of origin or centre of diversity. The region may have germplasm suitable for hybridization breeding such as Oceania, New Guinea, and Hainan Island [12, 61].

### No single model exclusively is suitable for all studied traits in taro

The Q-Q plot shows how well the null hypothesis fits without phenotypic association with SNP. The expected and observed distributions should overlap and most SNPs should be diagonal. Power et al. [62] reported that some SNP deviations may reflect expanded *p*-values ​​due to population structure, but very few deviates from the diagonal of a truly polygenic trait. Overall, the results showed that for all the properties investigated, there is no single method that can better detect population-confounding effects than other methods. However, given the overall performance of the five models, Blink appeared to have a slight advantage over the other models and was selected for subsequent evaluation of all GWAS models.

### GEA identified local adapted loci and candidate genes

**Fig. 6:**
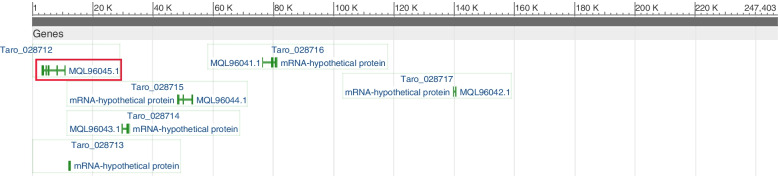
Graphic representation of candidate genes for *S_101024366* SNP marker region. The red colour is gene identified homologous region for astringency trait in *Diospyros lotus* (date-plum) and the green colours is other genes identified for the *S_101024366* SNP marker region

Signatures of selection and local adaptation can be evaluated in populations across entire genomes or genome sampling using population differentiation approaches (i.e., outliers) or in association with environmental variables to test the influence of biotic and abiotic factors in the spatial genomic structure. A total of nine SNP markers were associated with environmental variables. Specifically, *S_101024366* marker was significantly associated with all environmental variables except precipitation of the warmest quarter. The scaffold that contains this significant marker is *NMUH01001869.1* genebank accession number in NCBI. This accession region contains six candidate genes (Fig. 6). Hence, all the genes are six hypothetical unknown proteins in taro genome. The genes nucleotide sequences blasted in NCBI using default parameters. Hence, one of the genes, accession number *MQL96045.1* (*Taro_0284712*), identified the homologues region in *Diospyros lotus* (date-plum) DNA for the astringency trait with the *2e-15* E-values and 85.86% of identity. This *Taro_0284712* is in the range of LD window size (35 kb). One of the most essential aspects of fruit sensory quality is astringency [63, 64]. This might favour during human selection. Astringency is dominant in tannin sorghums [65]. Traditional sorghum varieties with medium tannin (moderate astringency) levels are widely cultivated and utilized for staple foods and alcoholic beverages in eastern and southern Africa [66]. However, some African cultures prefer tannin sorghums (more astringency) because the porridge from tannin sorghums stays in the stomach longer and giving the farmer the feeling of being full for the majority of the working day. Taro leaves are known by astringent due to the acridity content of the plant [65].

**Fig. 7:**
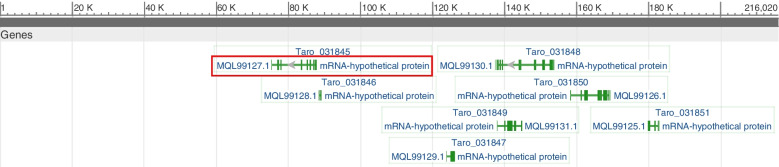
Graphic representation of candidate genes for *S_100991964* SNP marker region (*NMUH01002301.1*). The red, blue and yellow colour is gene identified homologous region for diaminopimelate decarboxylase gene, *Cyclin dependent kinases (CDKs)* and *MYB transcription factor (MYB),* respectively. The green colours are other genes identified for the S_100991964 SNP marker region still unknow protein

Another significantly associated marker is *S_100991964*. It was associated with all precipitation variables such as annual precipitation, precipitation of wettest quarter, precipitation of driest quarter, precipitation of warmest quarter, and precipitation of coldest quarter. *NMUH01002301.1* (*Colocasia esculenta cultivar Niue isolate Niue_2 TARO_scaffold_002301*) accession number or Scaffold contained this *S_100991964* SNP marker. Seven genes were linked within *NMUH01002301.1* accession which was identified as hypothetical protein in taro (Fig. 7). Again, the genes nucleotide sequences blasted in NCBI using default parameter. One of the genes (*MQL99127.1*, *Taro_031845*) is homologous with diaminopimelate decarboxylase gene in different crops (*Hevea brasiliensis, Gossypium arboretum, Manihot esculenta, Jatropha curcas, Ricinus communis, Populus alba,* and *Citrus sinensis*) with E-values *2e-37* to *4e-19*. Interestingly, this diaminopimelate decarboxylase gene is highly expressed under induced drought stress in different crops [67].

Another gene (*MQL99126.1*), co-located with *S_100991964* SNP marker, was found homologues with cyclin dependent kinase (CDK) gene in different plants (*Populus alba*, *Daucus carota*, *Prosopis alba*, *Zingiber officinale*, *Glycine max,* and *Brassica rapa*) with the E-values ranging *5e-08* to *4e-04*. CDKs are core cell cycle regulators and play important role in different aspects of plant growth and development [67, 68]. Several studies have indicated the involvement of *CDKs* in the plant stress responses [68–71]. Magwanga et al. [70] also reported that the possibility of *CDKF-4 s* and *CDKG-2 s* primary regulators of drought responses in cotton.

*MQL99125.1*gene, co-located with *S_100991964* SNP marker, has a homologues region with *MYB transcription factor (MYB)* in different plants (*Anthurium andraeanum, Elaeis guineensis, Ricinus communis, Pinus radiata, Triticum aestivum,* and *Hordeum vulgare*). *MYB* family transcription factors play crucial roles in response to abiotic stresses [72, 73]. For instance, *TaMYB31* is transcriptionally induced by drought stress in *Arabidopsis thaliana* [74].

### Genome-wide association study (GWAS)

Mapping traits in taro population provides another opportunity to validate and use the SNP markers for further breeding programs. GWAS identified a total of 45, 40 and 34 significant SNP markers associated with studied traits in combined, year 1 and year 2 data sets, respectively. Out of these, five markers were identified in two data sets out of the three, including S1_18891752 S3_100795476, S1_100584471 S1_100896936 and S2_100587991. Additionally, single SNP marker (*S2_100587991* SNP) was associated with a climatic variable (mean temperature of warmest quarter) and phenotypic trait (yield per hectare). *S2_100587991* SNP is found in scaffold of *NMUH01001840.1*. This scaffold contains 17 genes identified as hypothetical proteins in the taro genome. Several genes are linked to the identified five significant SNP markers that are identified as hypothetic proteins in the taro genome with the 35 kb window size. The Blast result is presented in detail in Table S5.

## Conclusion

Southeast Nigerian taro is high in phenotypic and genetic diversity with low admixture. This may be due to taro being an asexually propagated crop. The Nigerian taro diversity is less explained by the environment as other factors such as human activities might have a major role in taro diversity. Therefore, feasible strategy must be in place to encourage farmers to conserve the genetic resources. This study identified that genomic signatures of adaptation are useful for germplasm characterization, potentially enhancing future marker-assisted selection and taro crop improvement in Nigeria. These findings suggest that the allelic distribution at astringent, *CDK*, and *MYB* transcription factors might be shaped by geographical gradients in human and natural selection. However, further evaluation of the genes or genomic region is recommended.

## Supplementary Information

**Additional file 1: Table S1.** List of Nigerian Taro accessions with passport data.

**Additional file 2: Table S2.** Climatic data for BIO1 = Annual mean temperature (°C), BIO8 = Mean temperature of wettest quarter(°C) BIO9 = Mean temperature of driest quarter (°C), BIO10 = Mean temperature of warmest quarter (°C), BIO11 = Mean temperature of coldest quarter(°C), BIO12 = Annual precipitation(mm), BIO16 = Precipitation of wettest quarter(mm), BIO17(mm) = Precipitation of driest quarter (mm), BIO18 = Precipitation of warmest quarter (mm) and BIO19 = Precipitation of coldest quarter(mm).

**Additional file 3: Table S3.** BLUE mean values of phenotypic traits: COD = corm diameter (cm), COL = corm length (cm), CRD = cormel diameter (cm), CRL = cornel length (cm), CRW = cormel weight (g), DM = days to maturity, NCR = Number of cormels per plant, PH = plant height (cm), NLPP = number of leaves per plant, NSPP = number of suckers per plant, PL = petiole length (cm), YPH (t/ha) = yield per hectare and YPP = yield per plants (kg/plant).

**Additional file 4: Fig. S1.** Estimates of subpopulations analysis of 92 diverse taro landraces revealed 4 subpopulation using cross-entropy values with LEA R package program

**Additional file 5: Table S4.** Genomic Control Inflation Factor (λGC) analyses of GWAS models as a function of three different methods a for adjusting population structure on climatic variables and agro-morphological traits.

**Additional file 6: Fig. S2.** Q-Q plot of climatic variables (BIO1 = Annual mean temperature, BIO8 = Mean temperature of wettest quarter, BIO9 = Mean temperature of driest quarter, BIO10 = Mean temperature of warmest quarter, BIO11 = Mean temperature of coldest quarter, BIO12 = Annual precipitation, BIO16 = Precipitation of wettest quarter, BIO17 = Precipitation of driest quarter, BIO18 = Precipitation of warmest quarter and BIO19 = Precipitation of coldest quarter) with different models (Blink = Bayesian-information and Linkage-disequilibrium Iteratively Nested Keyway, CMLM = copressed mixed linear models, GLM = general linear model, MLM = mixed linear models, and FarmCPU = Fixed and random model Circulating Probability Unification).

**Additional file 7: Fig. S3.** Q-Q plots of phenotypic traits (COD = corm diameter (cm), COL = corm length (cm), CRD = cormel diameter (cm), CRL = cornel length (cm), CRW = cormel weight (g), DM = days to maturity, NCR = Number of cormels per plant, PH = plant height (cm), NLPP = number of leaves per plant, NSPP = number of suckers per plant, PL = petiole length (cm), YPH (t/ha) = yield per hectare and YPP = yield per plants (kg/plant) using different models (Blink = Bayesian-information and Linkage-disequilibrium Iteratively Nested Keyway, CMLM = copressed mixed linear models, GLM = general linear model, MLM = mixed linear models, and FarmCPU = Fixed and random model Circulating Probability Unification).

**Additional file 8: Fig. S4.** genome-environment association (GEA) across the Nigerian taro landrace collection using 9442 SNP markers (MFA ≥ 0.01). Manhattan plots showing significant false discovery rate (FDR) adjusted *P*-value of < 0.05 associated with climatic variables for climatic variables (BIO1 = Annual mean temperature, BIO8 = Mean temperature of wettest quarter, BIO9 = Mean temperature of driest quarter, BIO10 = Mean temperature of warmest quarter, BIO11 = Mean temperature of coldest quarter, BIO12 = Annual precipitation, BIO16 = Precipitation of wettest quarter, BIO17 = Precipitation of driest quarter, BIO18 = Precipitation of warmest quarter and BIO19 = Precipitation of coldest quarter). The x-axis represents the chromosomes and the y-axis the –log10 (*P*-values) for marker–environment association. Each point represents the SNP marker. The threshold is set based on the Genetic Type I error calculator (GEC) of the *P*-values.

**Additional file 9: Fig. S5.** A. Genome-wide association study across the Nagerian taro landrace collection using 9442 SNP markers (MFA ≥ 0.01) and combined data set. Manhattan plots showing significant false discovery rate (FDR) adjusted *P*-value of < 0.05 associated with phenotypic traits. The x-axis represents the chromosomes and the y-axis the –log10 (*P*-values) for marker–trait association. Each point represents the SNP marker. The threshold is set based on the Genetic Type I error calculator (GEC) of the *P*-values.

**Additional file 10: Table S5.** List of co-located genes around five significant markers of the traits: COD = corm diameter (cm), COL = corm length (cm), CRD = cormel diameter (cm), CRL = cornel length (cm), CRW = cormel weight (g), DM = days to maturity, NCR = Number of cormels per plant, PH = plant height (cm), NLPP = number of leaves per plant, NSPP = number of suckers per plant, PL = petiole length (cm), YPH (t/ha) = yield per hectare and YPP = yield per plants (kg/plant).

## Data Availability

All data generated or analysed during this study are included in this published article (and its supplementary information files). The sequencing data of 92 accessions used in this study have been deposited into the NCBI database under accession number PRJNA901400 (https://www.ncbi.nlm.nih.gov/bioproject/PRJNA901400).

## Abbreviations

BecA-ILRI: The Biosciences eastern and central Africa (BecA)-ILRI
Blink: Bayesian-information and Linkage-disequilibrium Iteratively Nested Keyway
CDK: Cyclin dependant kinase
CMLM: Compressed mixed linear model
DArTseq: Diversity Array Technology sequencing
EBSU: Ebonyi State University
ECV: environment coefficient of variation
GAPIT: Genome Association and Prediction Integrated Tool
GCV: Genetic coefficient of variation
GEA: Genome-environment association
GLM: General linear model
GWAS: Genome-environment association
IGSS: Integrated Genotyping Support and Service
LD: Linkage Disequilibrium
LEA: Landscape and Ecological Association Studies
LFMM: Latent Factor Mixed Models
MLM: Mixed linear model
NCBI: National Center for Biotechnology Information
NMF: Non-negative matrix factorization
PCA: Principal component analysis
PCV: Phenotype confident of variation
Q-Q plot: Quantile-quantile plot
RDA: Redundancy analysis
SE: Southeast
