# Supplementary material for: Detection of the local adaptive and genome-wide associated loci in southeast Nigerian taro (Colocasia esculenta (L.) Schott) populations
Source: BMC Genomics. 2023 Jan 24;24:39. doi: 10.1186/s12864-023-09134-6 (PMC9872430; doi:10.1186/s12864-023-09134-6)
Supplement: Supplementary file 1 — Additional file 1: Table S1. List of Nigerian Taro accessions with passport data. [file 12864_2023_9134_MOESM1_ESM.docx]

Table S1: List of Nigerian Taro accessions with passport data.

| No | Accessions | State | Altitude(m) | Longitude (N) | Latitude (E) |
| --- | --- | --- | --- | --- | --- |
| 1 | EBNFC001 | Ebonyi | 106 | 06^o^23.897’ | 007^o^58.342’ |
| 2 | EBNFC002 | Ebonyi | 106 | 06^o^23.897’ | 007^o^58.342’ |
| 3 | EBNFC003 | Ebonyi | 106 | 06^o^23.897’ | 007^o^58.342’ |
| 4 | EBNFC004 | Ebonyi | 66 | 06^o^12.653’ | 008^o^03.839’ |
| 5 | EBNFC005 | Ebonyi | 66 | 06^o^12.653’ | 008^o^03.839’ |
| 6 | EBNFC006 | Ebonyi | 55 | 06^o^12.909’ | 008^o^03.383’ |
| 7 | EBNFC007 | Ebonyi | 66 | 06^o^12.862’ | 008^o^03.928’ |
| 8 | EBNFC008 | Ebonyi | 66 | 06^o^12.862’ | 008^o^03.928’ |
| 9 | EBNFC009 | Ebonyi | 42 | 06^o^09.403’ | 008^o^08.369’ |
| 10 | EBNFC010 | Ebonyi | 75 | 06^o^10.137' | 008^o^01.773' |
| 11 | EBNFC011 | Ebonyi | 75 | 06^o^10.137' | 008^o^01.773' |
| 12 | EBNFC012 | Ebonyi | 75 | 06^o^10.137' | 008^o^01.773' |
| 13 | EBNFC013 | Ebonyi | 75 | 06^o^10.137' | 008^o^01.773' |
| 14 | EBNFC014 | Ebonyi | 75 | 06^o^10.137' | 008^o^01.773' |
| 15 | EBNFC015 | Ebonyi | 75 | 06^o^10.137' | 008^o^01.773' |
| 16 | EBNFC016 | Ebonyi | 75 | 06^o^10.137' | 008^o^01.773' |
| 17 | EBNFC017 | Ebonyi | 75 | 06^o^10.137' | 008^o^01.773' |
| 18 | EBNFC018 | Ebonyi | 75 | 06^o^10.137' | 008^o^01.773' |
| 19 | EBNFC019 | Ebonyi | 75 | 06^o^10.137' | 008^o^01.773' |
| 20 | EBNFC020 | Ebonyi | 75 | 06^o^10.137' | 008^o^01.773' |
| 21 | EBNFC021 | Ebonyi | 75 | 06^o^10.137' | 008^o^01.773' |
| 22 | EBNFC022 | Anambra | 121 | 06^o^09.351' | 006^o^52.873' |
| 23 | EBNFC023 | Anambra | 121 | 06^o^09.351' | 006^o^52.873' |
| 24 | EBNFC024 | Anambra | 121 | 06^o^09.351' | 006^o^52.873' |
| 25 | EBNFC025 | Anambra | 121 | 06^o^09.351' | 006^o^52.873' |
| 26 | EBNFC026 | Anambra | 121 | 06^o^09.351' | 006^o^52.873' |
| 27 | EBNFC027 | Anambra | 121 | 06^o^09.351' | 006^o^52.873' |
| 28 | EBNFC028 | Anambra | 121 | 06^o^09.351' | 006^o^52.873' |
| 29 | EBNFC029 | Anambra | 121 | 06^o^09.351' | 006^o^52.873' |
| 30 | EBNFC030 | Anambra | 121 | 06^o^09.351' | 006^o^52.873' |
| 31 | EBNFC031 | Anambra | 121 | 06^o^09.351' | 006^o^52.873' |
| 32 | EBNFC032 | Anambra | 121 | 06^o^09.351' | 006^o^52.873' |
| 33 | EBNFC033 | Anambra | 121 | 06^o^09.351' | 006^o^52.873' |
| 34 | EBNFC034 | Anambra | 121 | 06^o^09.351' | 006^o^52.873' |
| 35 | EBNFC035 | Anambra | 138 | 06^o^09.335' | 006^o^52.868' |
| 36 | EBNFC036 | Anambra | 132 | 06^o^09.273' | 006^o^52.808' |
| 37 | EBNFC037 | Anambra | 132 | 06^o^09.273' | 006^o^52.808' |
| 38 | EBNFC038 | Anambra | 132 | 06^o^09.273' | 006^o^52.808' |
| 39 | EBNFC039 | Anambra | 132 | 06^o^09.273' | 006^o^52.808' |
| 40 | EBNFC040 | Anambra | 132 | 06^o^09.273' | 006^o^52.808' |
| 41 | EBNFC041 | Anambra | 132 | 06^o^09.273' | 006^o^52.808' |
| 42 | EBNFC042 | Enugu | 421 | 06^o^51.531' | 007^o^27.478' |
| 43 | EBNFC043 | Enugu | 421 | 06^o^51.531' | 007^o^27.478' |
| 44 | EBNFC044 | Enugu | 421 | 06^o^51.531' | 007^o^27.478' |
| 45 | EBNFC045 | Enugu | 421 | 06^o^51.531' | 007^o^27.478' |
| 46 | EBNFC046 | Enugu | 421 | 06^o^51.531' | 007^o^27.478' |
| 47 | EBNFC047 | Enugu | 421 | 06^o^51.531' | 007^o^27.478' |
| 48 | EBNFC048 | Enugu | 421 | 06^o^51.531' | 007^o^27.478' |
| 49 | EBNFC049 | Enugu | 421 | 06^o^51.531' | 007^o^27.478' |
| 50 | EBNFC050 | Enugu | 421 | 06^o^51.531' | 007^o^27.478' |
| 51 | EBNFC051 | Enugu | 421 | 06^o^51.531' | 007^o^27.478' |
| 52 | EBNFC052 | Enugu | 421 | 06^o^51.531' | 007^o^27.478' |
| 53 | EBNFC053 | Enugu | 421 | 06^o^51.531' | 007^o^27.478' |
| 54 | EBNFC054 | Enugu | 421 | 06^o^51.531' | 007^o^27.478' |
| 55 | EBNFC055 | Enugu | 421 | 06^o^51.531' | 007^o^27.478' |
| 56 | EBNFC056 | Enugu | 421 | 06^o^51.531' | 007^o^27.478' |
| 57 | EBNFC057 | Enugu | 421 | 06^o^51.531' | 007^o^27.478' |
| 58 | EBNFC058 | Enugu | 425 | 06^o^51.461' | 007^o^27.449' |
| 59 | EBNFC059 | Enugu | 425 | 06^o^51.461' | 007^o^27.449' |
| 60 | EBNFC060 | Enugu | 425 | 06^o^51.461' | 007^o^27.449' |
| 61 | EBNFC061 | Enugu | 425 | 06^o^51.461' | 007^o^27.449' |
| 62 | EBNFC062 | Abia | 114 | 05^o^49.735' | 007^o^20.781' |
| 63 | EBNFC063 | Abia | 94 | 05^o^50.735' | 007^o^26.781' |
| 64 | EBNFC064 | Abia | 94 | 05^o^50.735' | 007^o^26.781' |
| 65 | EBNFC065 | Imo | 108 | 05^o^46.588' | 007^o^15.476' |
| 66 | EBNFC066 | Imo | 108 | 05^o^46.588' | 007^o^15.476' |
| 67 | EBNFC067 | Imo | 108 | 05^o^46.588' | 007^o^15.476' |
| 68 | EBNFC068 | Imo | 108 | 05^o^46.588' | 007^o^15.476' |
| 69 | EBNFC069 | Imo | 108 | 05^o^46.588' | 007^o^15.476' |
| 70 | EBNFC070 | Imo | 108 | 05^o^46.588' | 007^o^15.476' |
| 71 | EBNFC071 | Imo | 108 | 05^o^46.588' | 007^o^15.476' |
| 72 | EBNFC072 | Imo | 108 | 05^o^46.588' | 007^o^15.476' |
| 73 | EBNFC073 | Imo | 149 | 05^o^52.552' | 007^o^18.135' |
| 74 | EBNFC074 | Imo | 166 | 05^o^49.506' | 007^o^21.010' |
| 75 | EBNFC075 | Imo | 163 | 05^o^49.506' | 007^o^20.993' |
| 76 | EBNFC076 | Imo | 167 | 05^o^49.506' | 007^o^18.979' |
| 77 | EBNFC077 | Abia | 94 | 05^o^50.735' | 007^o^26.781' |
| 78 | EBNFC078 | Imo | 167 | 05^o^49.534' | 007^o^20.975' |
| 79 | EBNFC079 | Imo | 167 | 05^o^49.533' | 007^o^20.975' |
| 80 | EBNFC080 | Imo | 167 | 05^o^49.533' | 007^o^20.975' |
| 81 | EBNFC081 | Imo | 167 | 05^o^49.533' | 007^o^20.975' |
| 82 | EBNFC082 | Imo | 167 | 05^o^49.533' | 007o20.975' |
| 83 | EBNFC083 | Imo | 167 | 05^o^49.533' | 007^o^20.975' |
| 84 | EBNFC084 | Imo | 167 | 05^o^49.533' | 007^o^20.975' |
| 85 | EBNFC085 | Imo | 167 | 05^o^49.533' | 007^o^20.975' |
| 86 | EBNFC086 | Abia | 94 | 05^o^50.735' | 007^o^26.781' |
| 87 | EBNFC087 | Abia | 94 | 05^o^50.735' | 007^o^26.781' |
| 88 | EBNFC088 | Abia | 94 | 05^o^50.735' | 007^o^26.781' |
| 89 | EBNFC089 | Abia | 94 | 05^o^50.735' | 007^o^26.781' |
| 90 | EBNFC090 | Abia | 94 | 05^o^50.735' | 007^o^26.781' |
| 91 | EBNFC091 | Abia | 94 | 05^o^50.735' | 007^o^26.781' |
| 92 | EBNFC092 | Abia | 94 | 05^o^50.735' | 007^o^26.781' |
| 93 | EBNFC093 | Abia | 94 | 05^o^50.735' | 007^o^26.781' |
| 94 | EBNFC094 | Abia | 94 | 05^o^50.735' | 007^o^26.781' |
| 95 | EBNFC095 | Abia | 94 | 05^o^50.735' | 007^o^26.781' |
| 96 | EBNFC096 | Abia | 94 | 05^o^50.735' | 007^o^26.781' |
| 97 | EBNFC097 | Abia | 94 | 05^o^50.735' | 007^o^26.781' |
| 98 | EBNFC098 | Abia | 94 | 05^o^50.735' | 007^o^26.781' |
| 99 | EBNFC099 | Abia | 94 | 05^o^50.735' | 007^o^26.781' |
| 100 | EBNFC100 | Abia | 94 | 05^o^50.735' | 007^o^26.781' |
