# Supplementary material for: Detection of the local adaptive and genome-wide associated loci in southeast Nigerian taro (Colocasia esculenta (L.) Schott) populations
Source: BMC Genomics. 2023 Jan 24;24:39. doi: 10.1186/s12864-023-09134-6 (PMC9872430; doi:10.1186/s12864-023-09134-6)

**Figure S1:** Estimates of subpopulations analysis of 92 diverse taro landraces revealed 4 subpopulation using cross-entropy values with LEA R package program


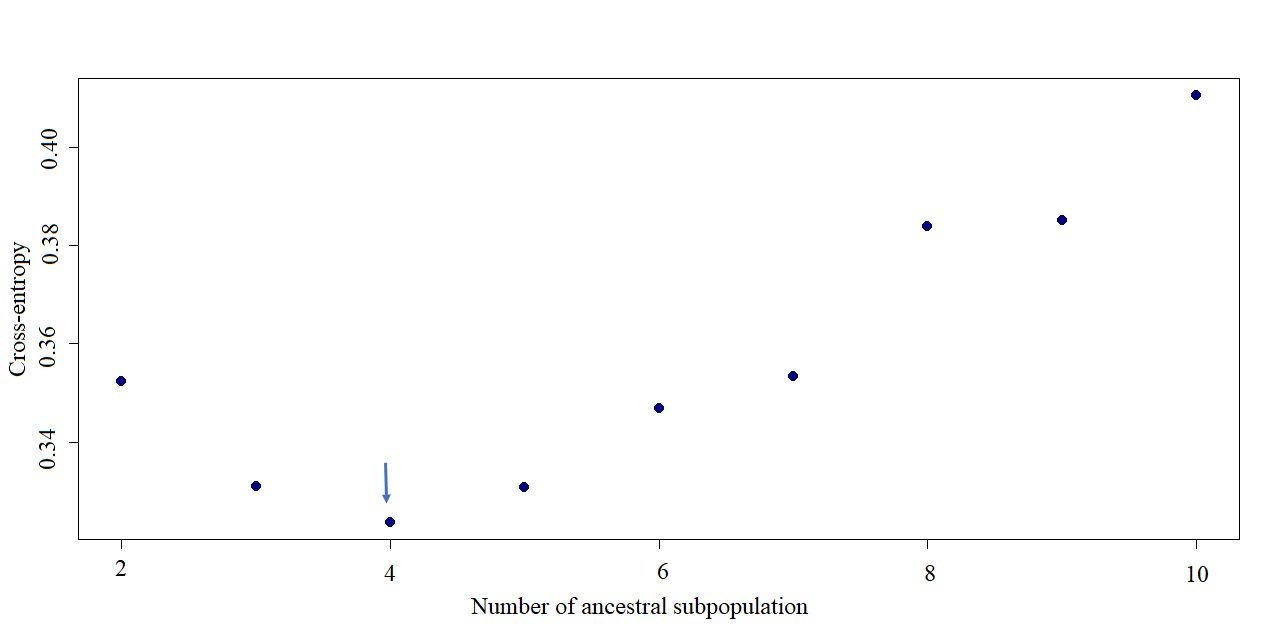

Supplement: Supplementary file 4 — Additional file 4: Fig. S1. Estimates of subpopulations analysis of 92 diverse taro landraces revealed 4 subpopulation using cross-entropy values with LEA R package program [file 12864_2023_9134_MOESM4_ESM.docx]
