# Supplementary material for: Detection of the local adaptive and genome-wide associated loci in southeast Nigerian taro (Colocasia esculenta (L.) Schott) populations
Source: BMC Genomics. 2023 Jan 24;24:39. doi: 10.1186/s12864-023-09134-6 (PMC9872430; doi:10.1186/s12864-023-09134-6)
Supplement: Supplementary file 5 — Additional file 5: Table S4. Genomic Control Inflation Factor (λGC) analyses of GWAS models as a function of three different methods a for adjusting population structure on climatic variables and agro-morphological traits. [file 12864_2023_9134_MOESM5_ESM.docx]

Table S4: Genomic Control Inflation Factor (λGC) analyses of GWAS models as a function of three different methods a for adjusting population structure on climatic variables and agro-morphological traits.

| Variables | | Genomic inflation factor (λ_GC)_ | | | | | Rank^a^ | | | | |
| --- | --- | --- | --- | --- | --- | --- | --- | --- | --- | --- | --- |
|  |  | GLM | MLM | FarmCPU | CMLM | BLINK | GLM | MLM | FarmCPU | CMLM | BLINK |
| Climatic | BIO1 | 0.9807 | 0.9812 | 0.9807 | 0.9429 | 0.9824 | 3 | 2 | 3 | 4 | 1 |
|  | BIO8 | 0.9475 | 0.9537 | 0.9475 | 0.9244 | 0.9537 | 2 | 1 | 2 | 3 | 1 |
|  | BIO9 | 0.9607 | 0.9669 | 0.9607 | 0.9067 | 0.9577 | 2 | 1 | 2 | 4 | 3 |
|  | BIO10 | 1.0353 | 0.9839 | 1.0353 | 0.9836 | 0.9667 | 4 | 1 | 4 | 2 | 3 |
|  | BIO11 | 0.9475 | 0.9537 | 0.9475 | 0.9244 | 0.9537 | 2 | 1 | 2 | 3 | 1 |
|  | BIO12 | 0.8749 | 0.8809 | 0.8749 | 0.8870 | 1.0645 | 4 | 3 | 4 | 2 | 1 |
|  | BIO16 | 0.8986 | 0.9047 | 0.8986 | 0.9079 | 1.0490 | 4 | 3 | 4 | 2 | 1 |
|  | BIO17 | 0.8806 | 0.8867 | 0.8806 | 0.8998 | 1.0965 | 4 | 3 | 4 | 2 | 1 |
|  | BIO18 | 0.9145 | 0.9206 | 0.9145 | 0.9321 | 1.1090 | 3 | 2 | 3 | 1 | 4 |
|  | BIO19 | 0.8986 | 0.9047 | 0.8986 | 0.9079 | 1.0490 | 4 | 3 | 4 | 2 | 1 |
| Phenotype | COD | 1.1409 | 0.8930 | 1.1409 | 0.9282 | 0.9751 | 4 | 3 | 4 | 2 | 1 |
|  | COL | 0.9201 | 0.8987 | 0.9201 | 0.9203 | 0.9302 | 4 | 3 | 4 | 1 | 2 |
|  | CRD | 0.7913 | 0.7971 | 0.7913 | 0.7671 | 0.9955 | 3 | 2 | 3 | 4 | 1 |
|  | CRL | 0.9225 | 0.9288 | 0.9225 | 0.8731 | 0.8883 | 2 | 1 | 2 | 4 | 3 |
|  | CRW | 0.9874 | 0.9932 | 0.9874 | 0.9488 | 1.0391 | 2 | 1 | 2 | 4 | 3 |
|  | DM | 0.9790 | 0.9655 | 0.9790 | 0.9067 | 0.9099 | 1 | 2 | 1 | 4 | 3 |
|  | NCR | 1.0940 | 1.0382 | 1.0940 | 1.0180 | 0.9396 | 4 | 2 | 4 | 1 | 3 |
|  | NLPP | 1.1493 | 1.0553 | 1.1493 | 0.9913 | 1.0753 | 4 | 2 | 4 | 1 | 3 |
|  | NSPP | 1.1308 | 1.0271 | 1.1308 | 1.0558 | 1.0506 | 4 | 1 | 4 | 3 | 2 |
|  | PH | 0.9528 | 0.9589 | 0.9528 | 0.9377 | 1.0043 | 3 | 2 | 3 | 4 | 1 |
|  | PL | 1.1031 | 0.9982 | 1.1031 | 0.9035 | 0.9838 | 4 | 1 | 4 | 3 | 2 |
|  | YPH | 1.1113 | 0.9836 | 1.1113 | 1.0329 | 1.0619 | 4 | 1 | 4 | 2 | 3 |
|  | YPP | 1.1926 | 1.0641 | 1.1926 | 1.1110 | 1.1056 | 4 | 1 | 4 | 3 | 2 |

^a^=1=first; 2=second; 3=third and 4=forth; for cases where λ_GC_ was deflated (below or above 1), models closest to ‘1’ were ranked highest, BIO1 = Annual Mean Temperature, BIO8 = Mean Temperature of Wettest Quarter, BIO9 = Mean Temperature of Driest Quarter, BIO10 = Mean Temperature of Warmest Quarter, BIO11 = Mean Temperature of Coldest Quarter, BIO12 = Annual Precipitation, BIO16 = Precipitation of Wettest Quarter, BIO17 = Precipitation of Driest Quarter, BIO18 = Precipitation of Warmest Quarter and BIO19 = Precipitation of Coldest Quarter, COD= corm diameter (cm), COL= corm length (cm), CRD= cornel diameter 9cm), CRL=cornel length (cm), CRW= cornel weight (g), DM= dry matter, NCR= Number of cormels per plant, PH= plant height (cm), NLPP= number of leaves per plant, NSPP= number of suckers per plant, PL= petiole length (cm), YPH (t/ha)= yield per hectare, and YPP= yield per plants (kg/plant
