# Supplementary material for: Detection of the local adaptive and genome-wide associated loci in southeast Nigerian taro (Colocasia esculenta (L.) Schott) populations
Source: BMC Genomics. 2023 Jan 24;24:39. doi: 10.1186/s12864-023-09134-6 (PMC9872430; doi:10.1186/s12864-023-09134-6)

**Figure S2**: Q-Q plot of climatic variables (BIO1 = Annual mean temperature, BIO8 = Mean temperature of wettest quarter, BIO9 = Mean temperature of driest quarter, BIO10 = Mean temperature of warmest quarter, BIO11 = Mean temperature of coldest quarter, BIO12 = Annual precipitation, BIO16 = Precipitation of wettest quarter, BIO17 = Precipitation of driest quarter, BIO18 = Precipitation of warmest quarter and BIO19 = Precipitation of coldest quarter) with different models (Blink=Bayesian-information and Linkage-disequilibrium Iteratively Nested Keyway, CMLM=copressed mixed linear models, GLM= general linear model, MLM= mixed linear models, and FarmCPU= Fixed and random model Circulating Probability Unification)


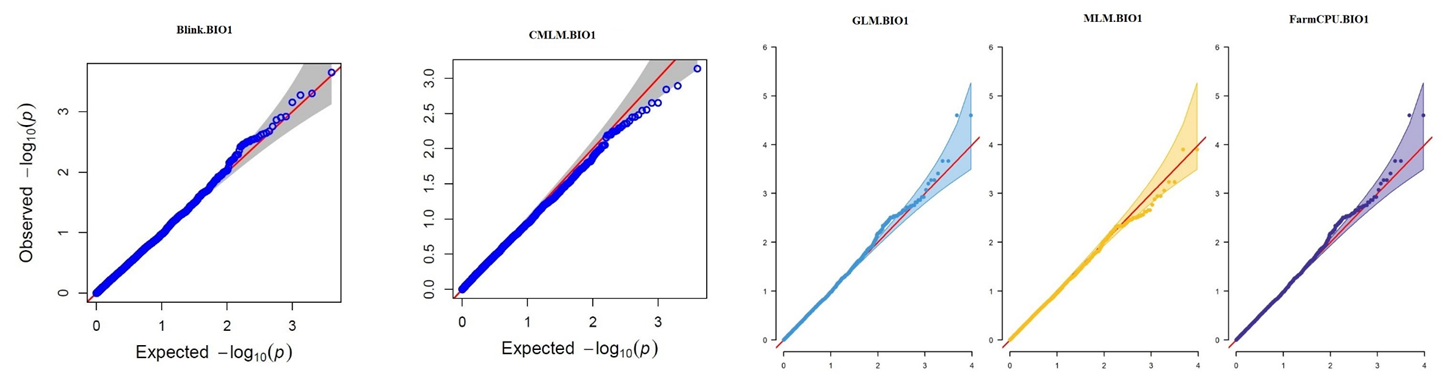


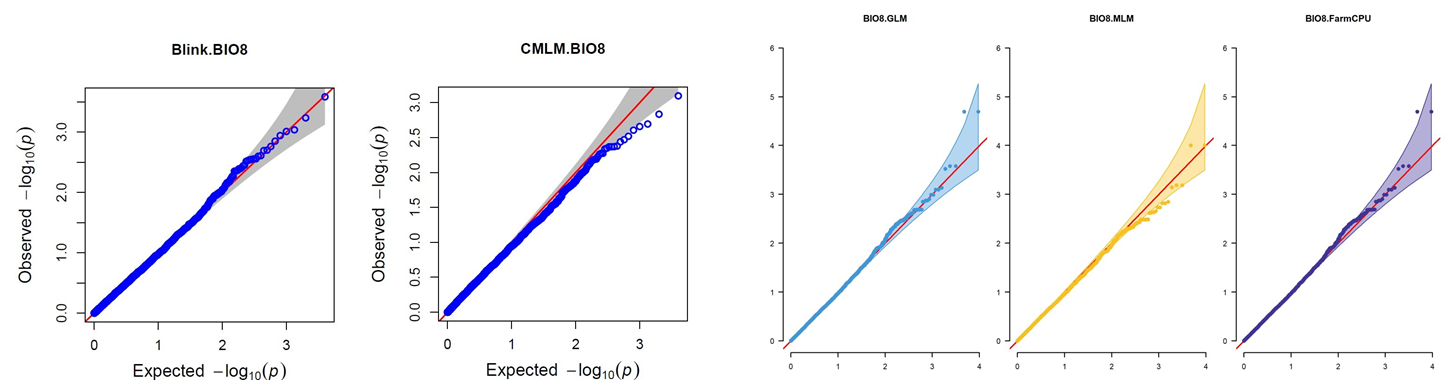


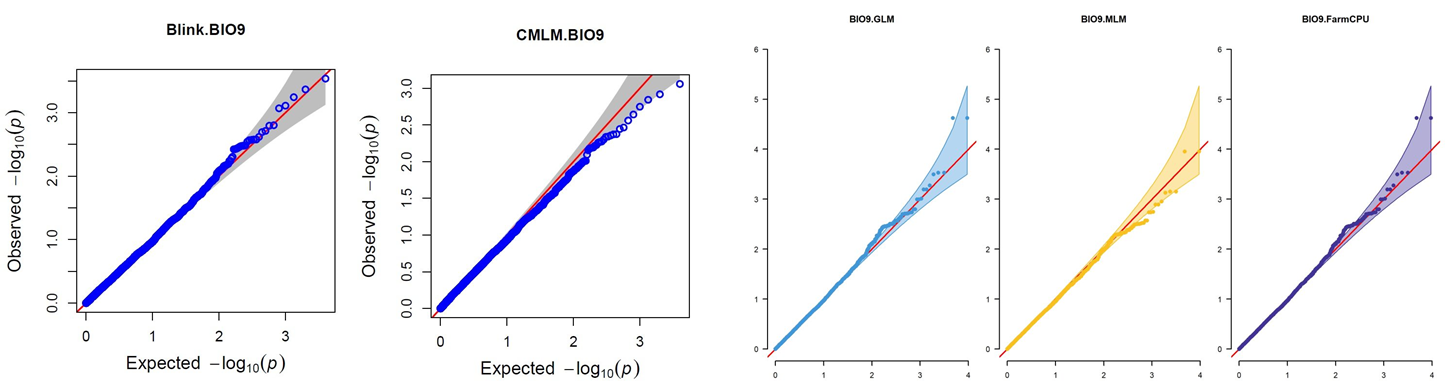


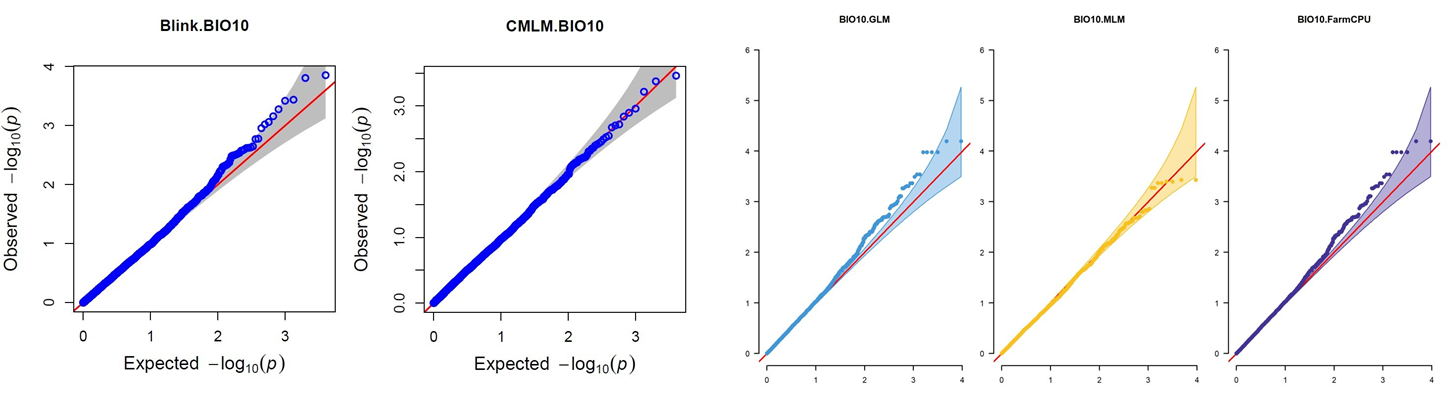


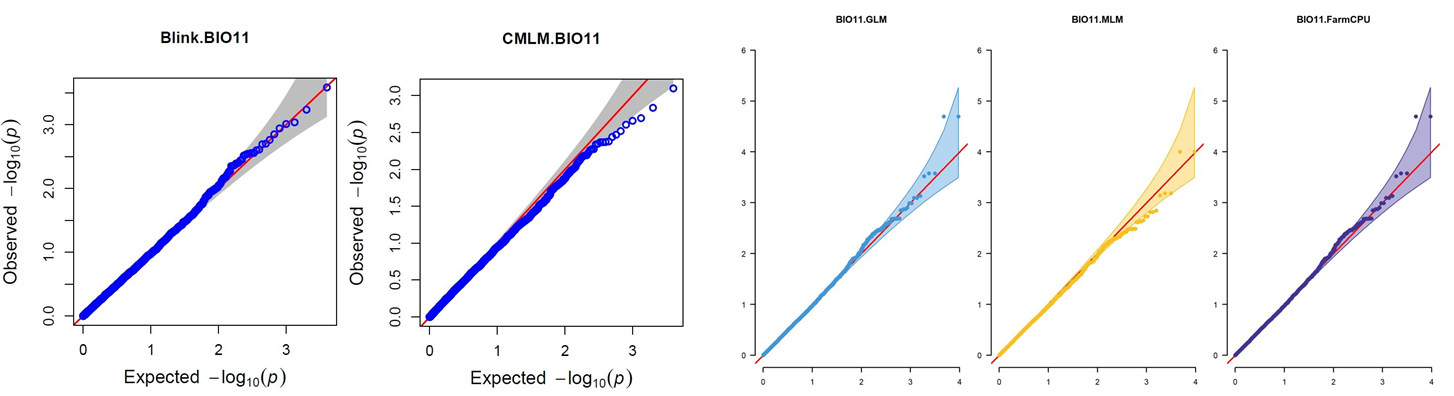


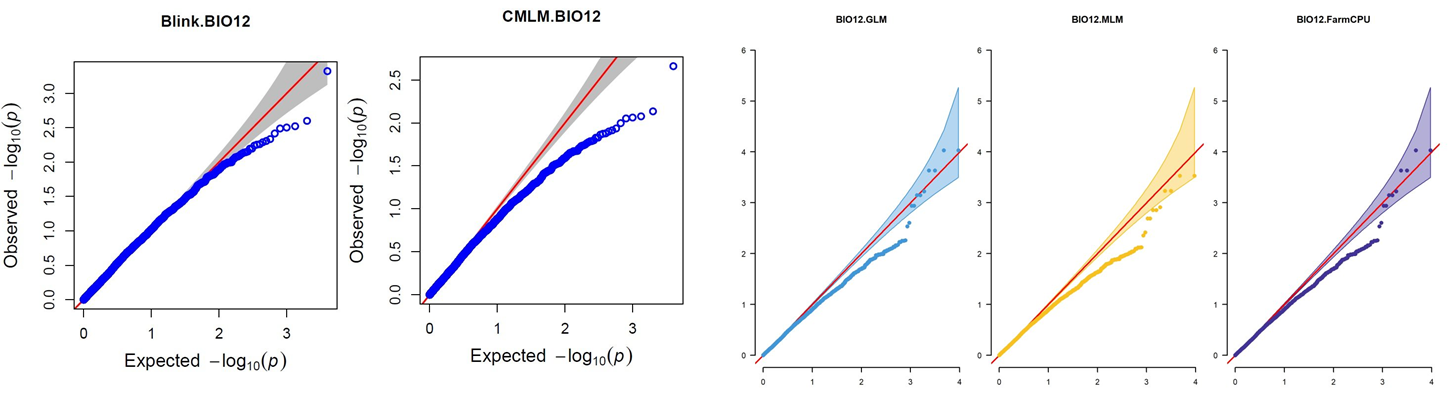


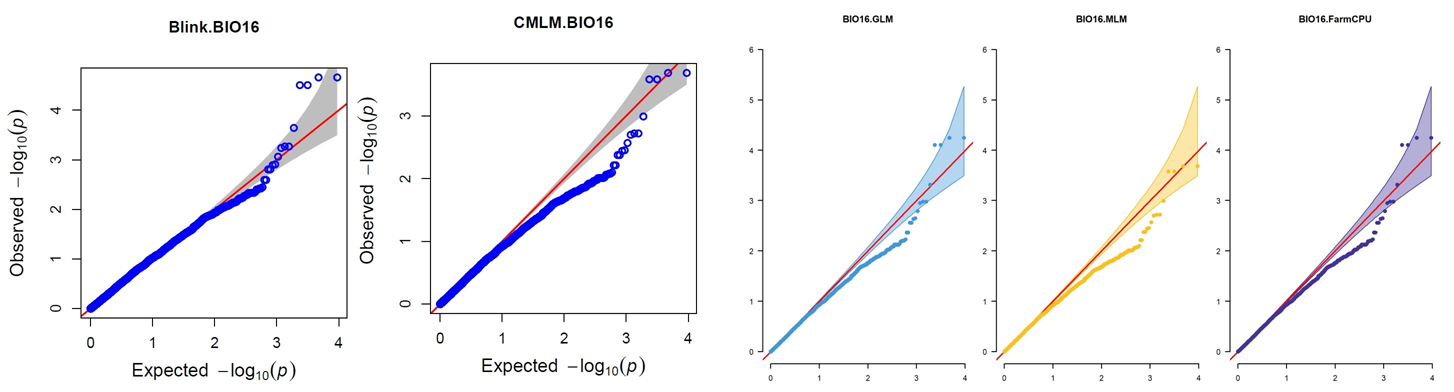


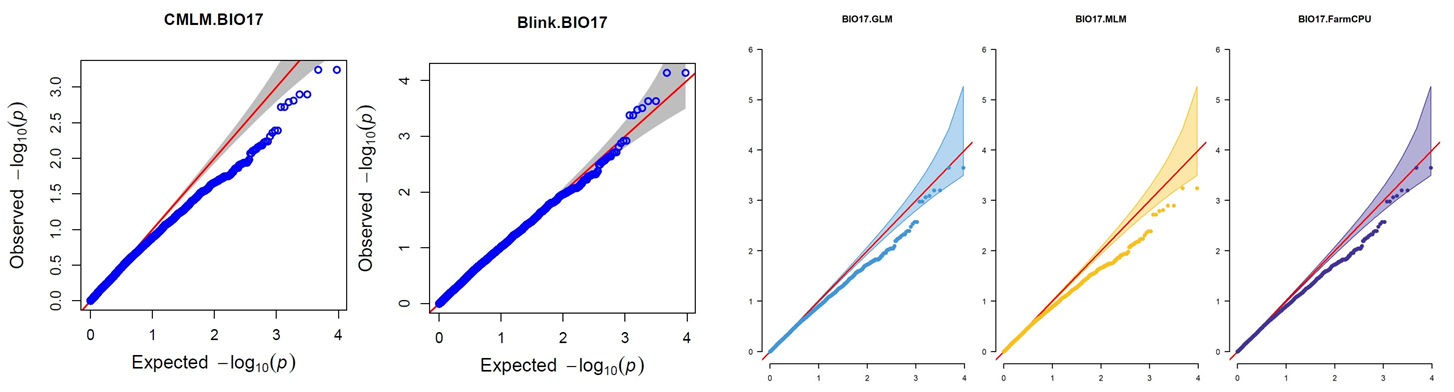


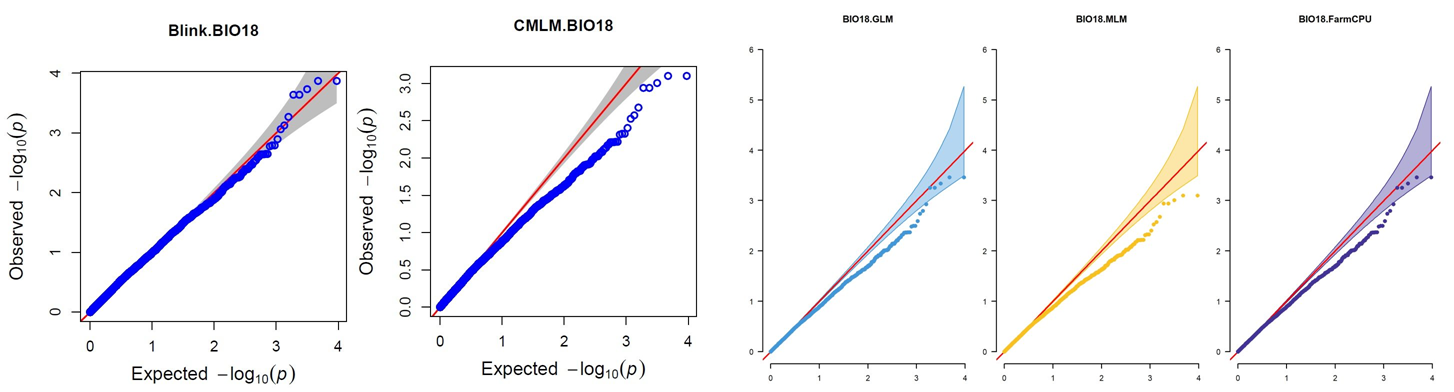


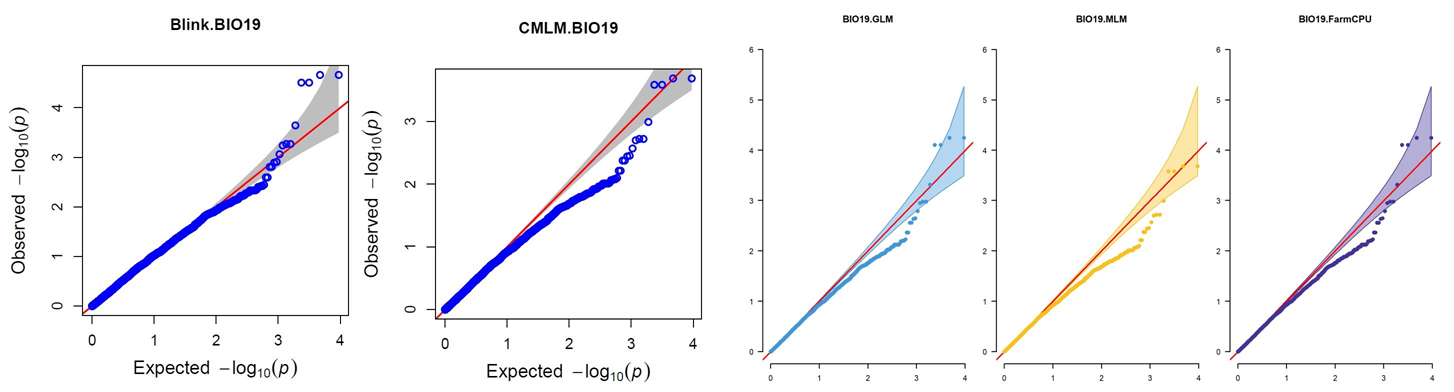

Supplement: Supplementary file 6 — Additional file 6: Fig. S2. Q-Q plot of climatic variables (BIO1 = Annual mean temperature, BIO8 = Mean temperature of wettest quarter, BIO9 = Mean temperature of driest quarter, BIO10 = Mean temperature of warmest quarter, BIO11 = Mean temperature of coldest quarter, BIO12 = Annual precipitation, BIO16 = Precipitation of wettest quarter, BIO17 = Precipitation of driest quarter, BIO18 = Precipitation of warmest quarter and BIO19 = Precipitation of coldest quarter) with different models (Blink = Bayesian-information and Linkage-disequilibrium Iteratively Nested Keyway, CMLM = copressed mixed linear models, GLM = general linear model, MLM = mixed linear models, and FarmCPU = Fixed and random model Circulating Probability Unification). [file 12864_2023_9134_MOESM6_ESM.docx]
