# Supplementary material for: Detection of the local adaptive and genome-wide associated loci in southeast Nigerian taro (Colocasia esculenta (L.) Schott) populations
Source: BMC Genomics. 2023 Jan 24;24:39. doi: 10.1186/s12864-023-09134-6 (PMC9872430; doi:10.1186/s12864-023-09134-6)

**Figure S3:** Q-Q plots of phenotypic traits (COD= corm diameter (cm), COL= corm length (cm), CRD= cormel diameter (cm), CRL=cornel length (cm), CRW= cormel weight (g), DM= days to maturity, NCR= Number of cormels per plant , PH= plant height (cm), NLPP= number of leaves per plant, NSPP= number of suckers per plant, PL= petiole length (cm) , YPH (t/ha)= yield per hectare and YPP= yield per plants (kg/plant) using different models (Blink=Bayesian-information and Linkage-disequilibrium Iteratively Nested Keyway, CMLM=copressed mixed linear models, GLM= general linear model, MLM= mixed linear models, and FarmCPU= Fixed and random model Circulating Probability Unification).


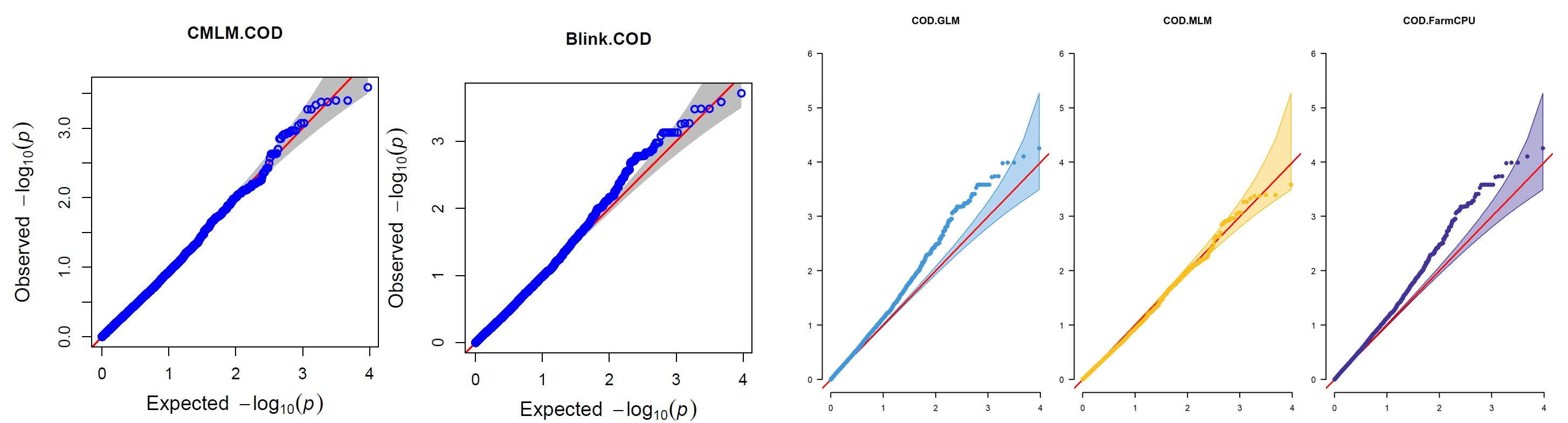

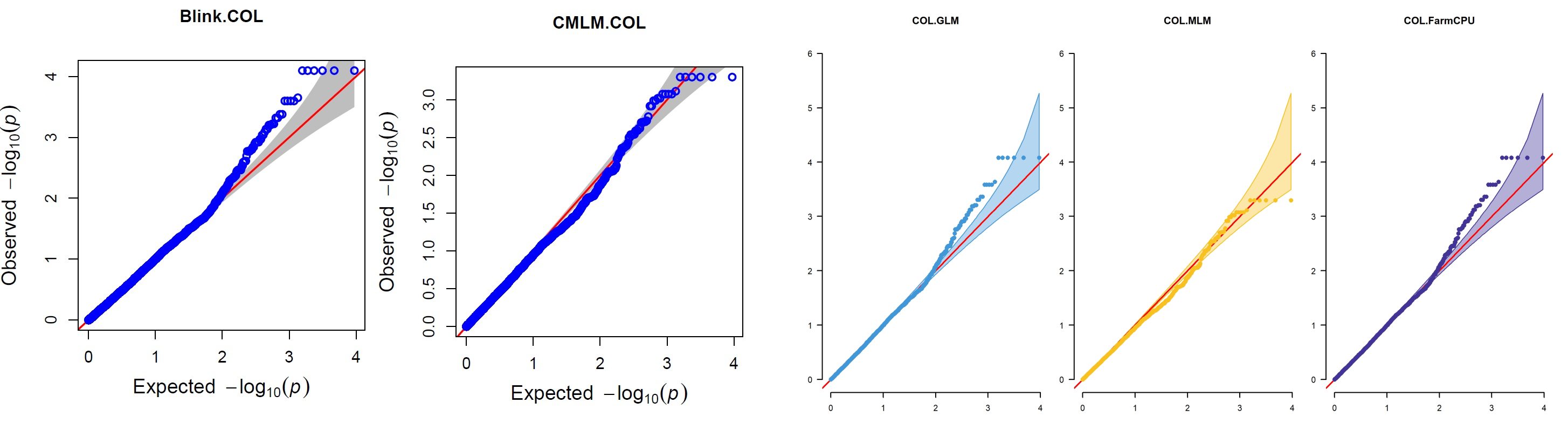


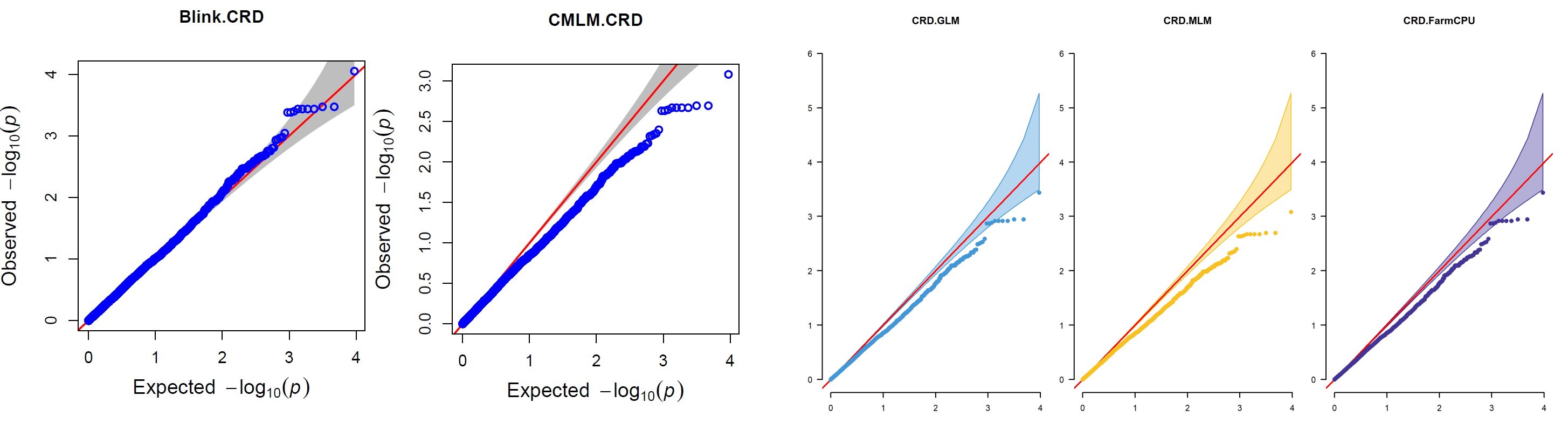

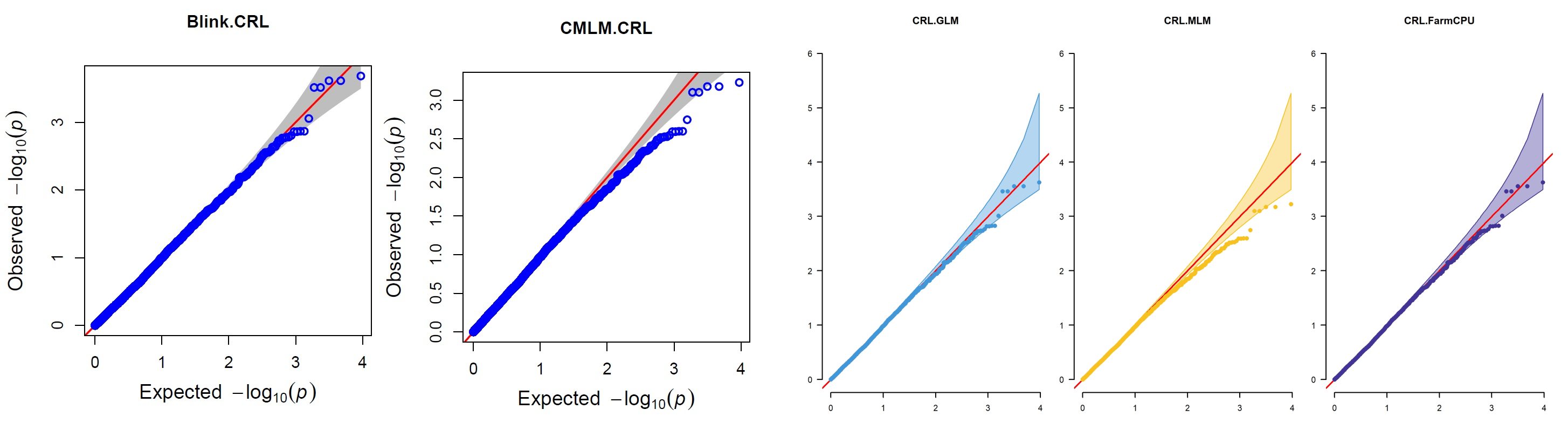


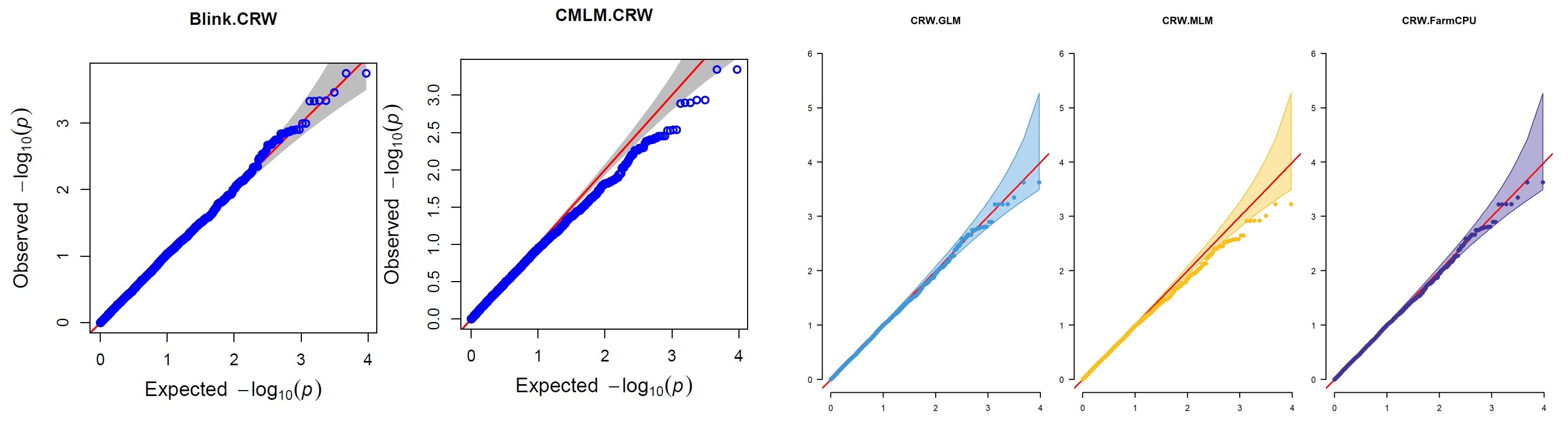

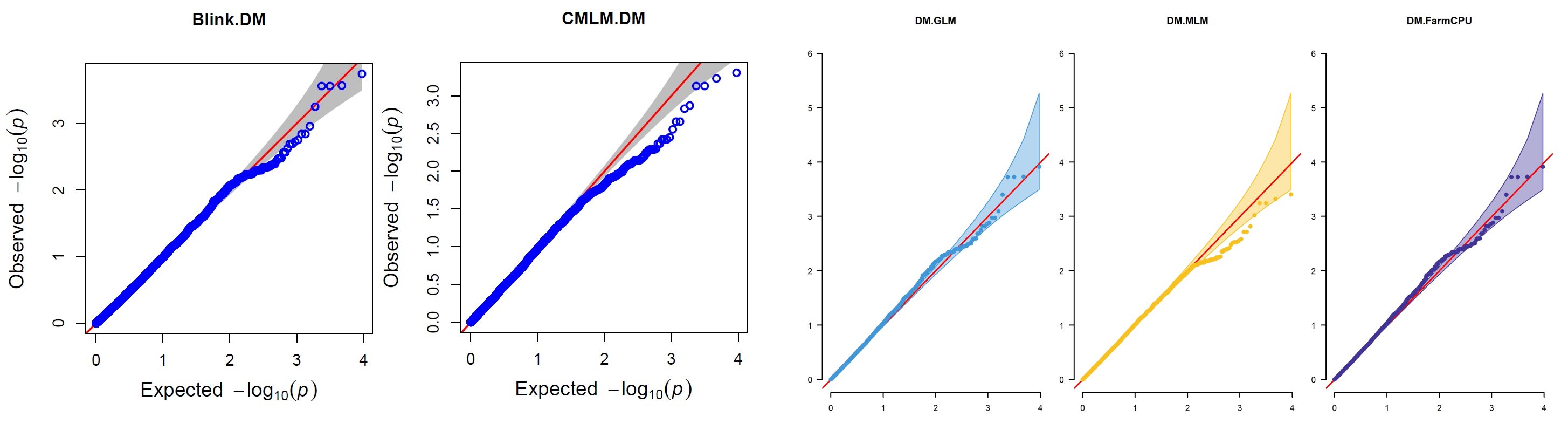


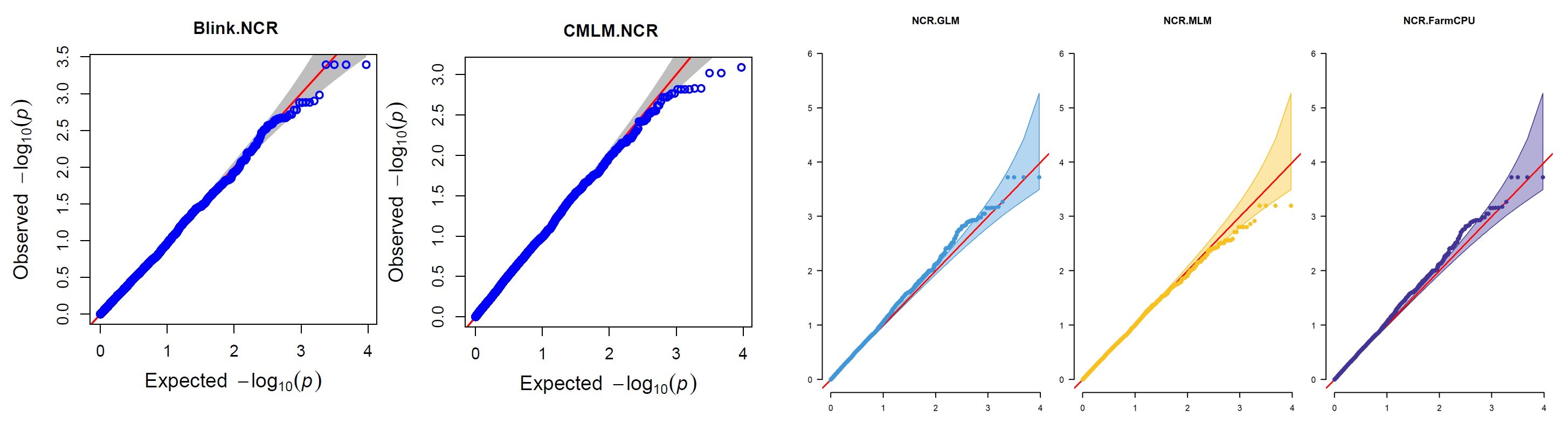

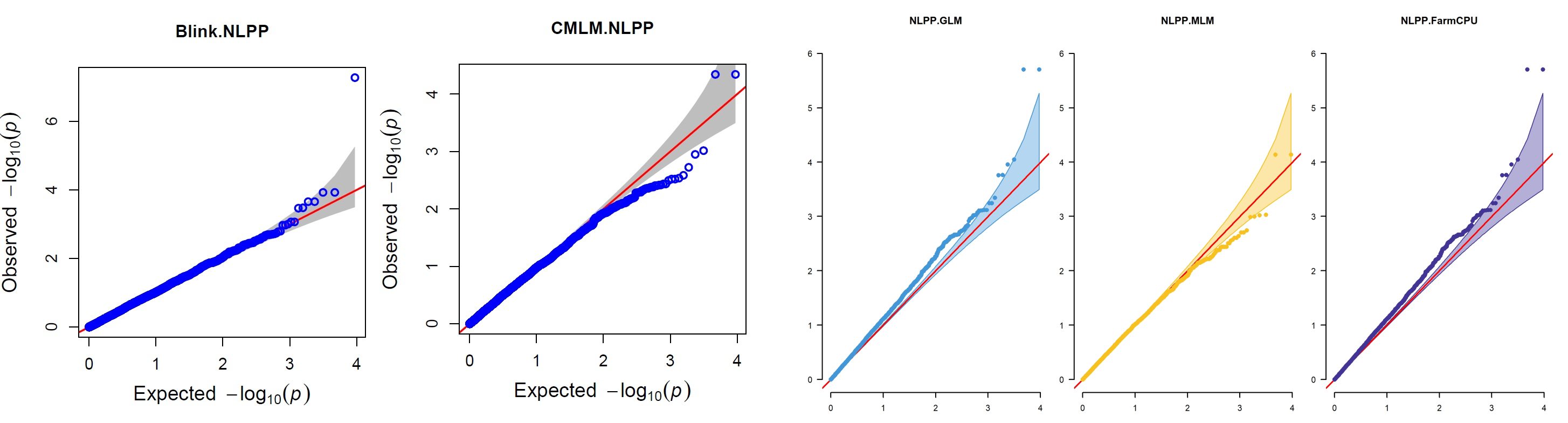


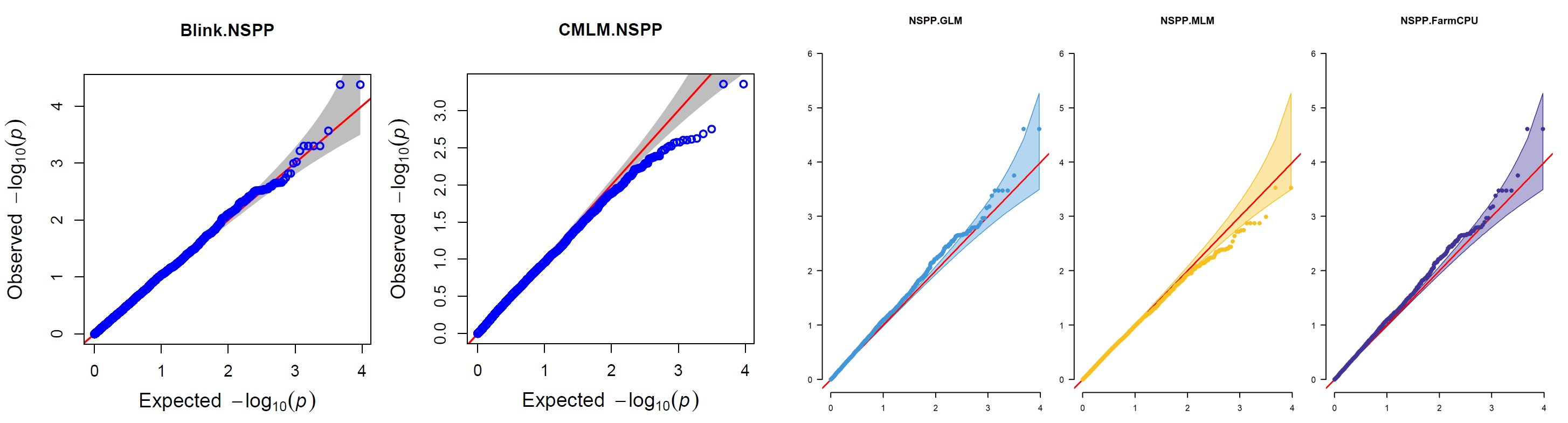

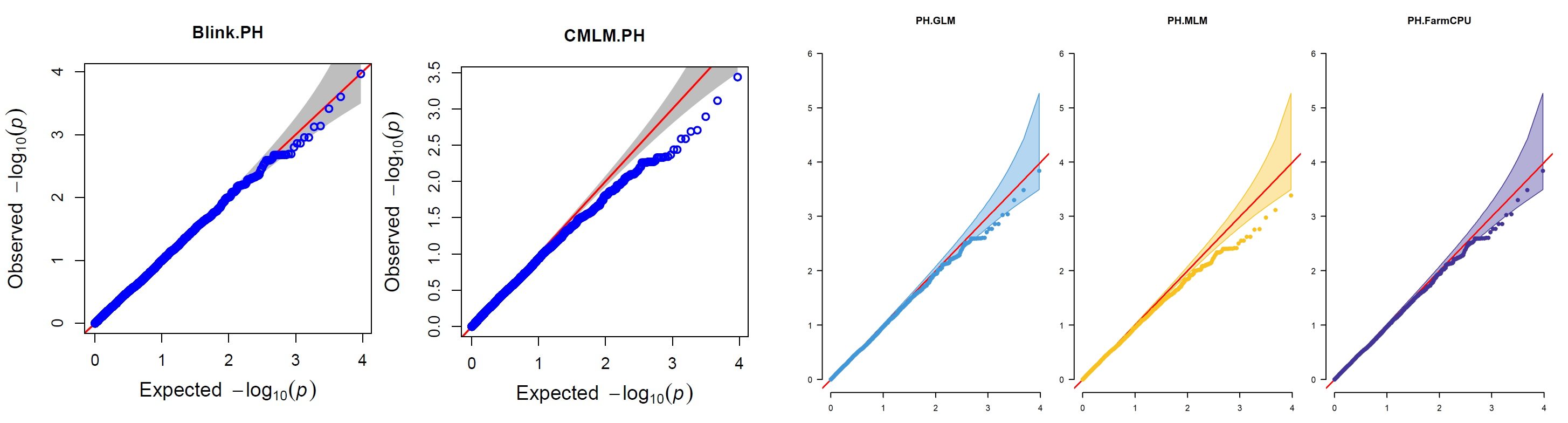


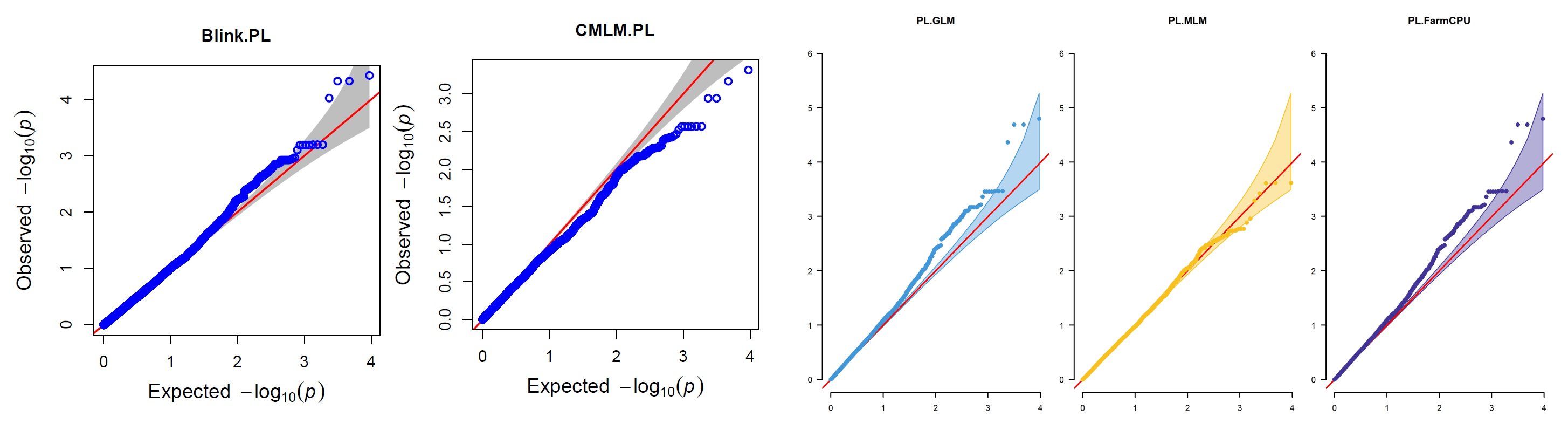


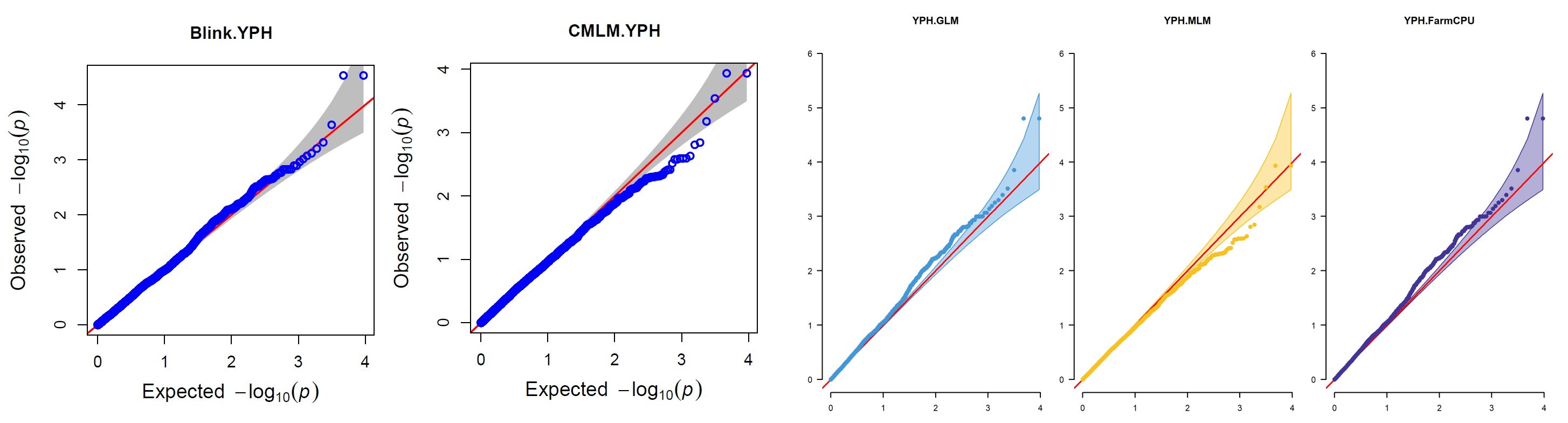


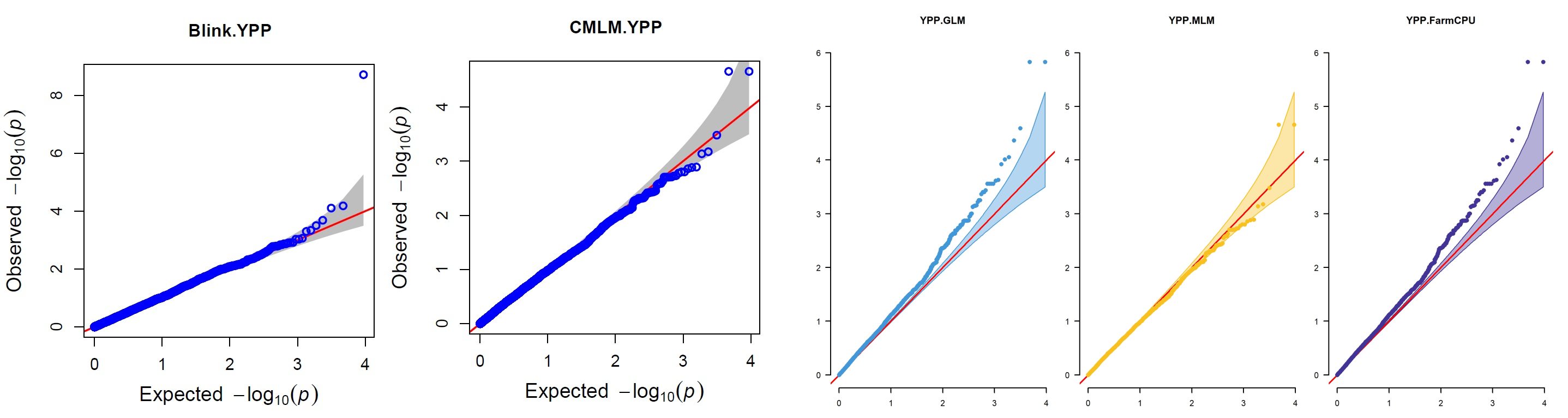

Supplement: Supplementary file 7 — Additional file 7: Fig. S3. Q-Q plots of phenotypic traits (COD = corm diameter (cm), COL = corm length (cm), CRD = cormel diameter (cm), CRL = cornel length (cm), CRW = cormel weight (g), DM = days to maturity, NCR = Number of cormels per plant, PH = plant height (cm), NLPP = number of leaves per plant, NSPP = number of suckers per plant, PL = petiole length (cm), YPH (t/ha) = yield per hectare and YPP = yield per plants (kg/plant) using different models (Blink = Bayesian-information and Linkage-disequilibrium Iteratively Nested Keyway, CMLM = copressed mixed linear models, GLM = general linear model, MLM = mixed linear models, and FarmCPU = Fixed and random model Circulating Probability Unification). [file 12864_2023_9134_MOESM7_ESM.docx]
