# Supplementary material for: Detection of the local adaptive and genome-wide associated loci in southeast Nigerian taro (Colocasia esculenta (L.) Schott) populations
Source: BMC Genomics. 2023 Jan 24;24:39. doi: 10.1186/s12864-023-09134-6 (PMC9872430; doi:10.1186/s12864-023-09134-6)

**Figure S4**: genome-environment association (GEA) across the Nigerian taro landrace collection using 9442 SNP markers (MFA≥0.01). Manhattan plots showing significant false discovery rate (FDR) adjusted P-value of <0.05 associated with climatic variables for climatic variables (BIO1 = Annual mean temperature, BIO8 = Mean temperature of wettest quarter, BIO9 = Mean temperature of driest quarter, BIO10 = Mean temperature of warmest quarter, BIO11 = Mean temperature of coldest quarter, BIO12 = Annual precipitation, BIO16 = Precipitation of wettest quarter, BIO17 = Precipitation of driest quarter, BIO18 = Precipitation of warmest quarter and BIO19 = Precipitation of coldest quarter). The x-axis represents the chromosomes and the y-axis the –log10 (P-values) for marker–environment association. Each point represents the SNP marker. The threshold is set based on the Genetic Type I error calculator (GEC) of the P-values.


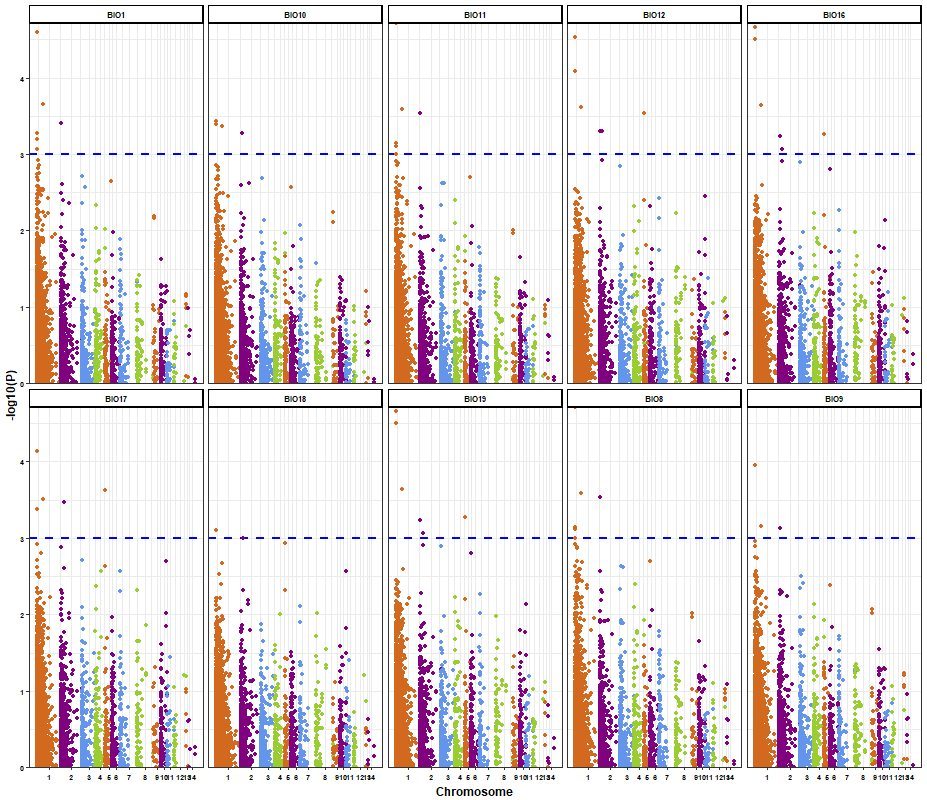

Supplement: Supplementary file 8 — Additional file 8: Fig. S4. genome-environment association (GEA) across the Nigerian taro landrace collection using 9442 SNP markers (MFA ≥ 0.01). Manhattan plots showing significant false discovery rate (FDR) adjusted P-value of < 0.05 associated with climatic variables for climatic variables (BIO1 = Annual mean temperature, BIO8 = Mean temperature of wettest quarter, BIO9 = Mean temperature of driest quarter, BIO10 = Mean temperature of warmest quarter, BIO11 = Mean temperature of coldest quarter, BIO12 = Annual precipitation, BIO16 = Precipitation of wettest quarter, BIO17 = Precipitation of driest quarter, BIO18 = Precipitation of warmest quarter and BIO19 = Precipitation of coldest quarter). The x-axis represents the chromosomes and the y-axis the –log10 (P-values) for marker–environment association. Each point represents the SNP marker. The threshold is set based on the Genetic Type I error calculator (GEC) of the P-values. [file 12864_2023_9134_MOESM8_ESM.docx]
