# Supplementary material for: Detection of the local adaptive and genome-wide associated loci in southeast Nigerian taro (Colocasia esculenta (L.) Schott) populations
Source: BMC Genomics. 2023 Jan 24;24:39. doi: 10.1186/s12864-023-09134-6 (PMC9872430; doi:10.1186/s12864-023-09134-6)
Supplement: Supplementary file 9 — Additional file 9: Fig. S5. A. Genome-wide association study across the Nagerian taro landrace collection using 9442 SNP markers (MFA ≥ 0.01) and combined data set. Manhattan plots showing significant false discovery rate (FDR) adjusted P-value of < 0.05 associated with phenotypic traits. The x-axis represents the chromosomes and the y-axis the –log10 (P-values) for marker–trait association. Each point represents the SNP marker. The threshold is set based on the Genetic Type I error calculator (GEC) of the P-values. [file 12864_2023_9134_MOESM9_ESM.docx]

**Figure S5-A:** Genome-wide association study across the Nagerian taro landrace collection using 9,442 SNP markers (MFA≥0.01) and **combined data set**. Manhattan plots showing significant false discovery rate (FDR) adjusted P-value of <0.05 associated with phenotypic traits. The x-axis represents the chromosomes and the y-axis the –log10 (P-values) for marker–trait association. Each point represents the SNP marker. The threshold is set based on the Genetic Type I error calculator (GEC) of the P-values.


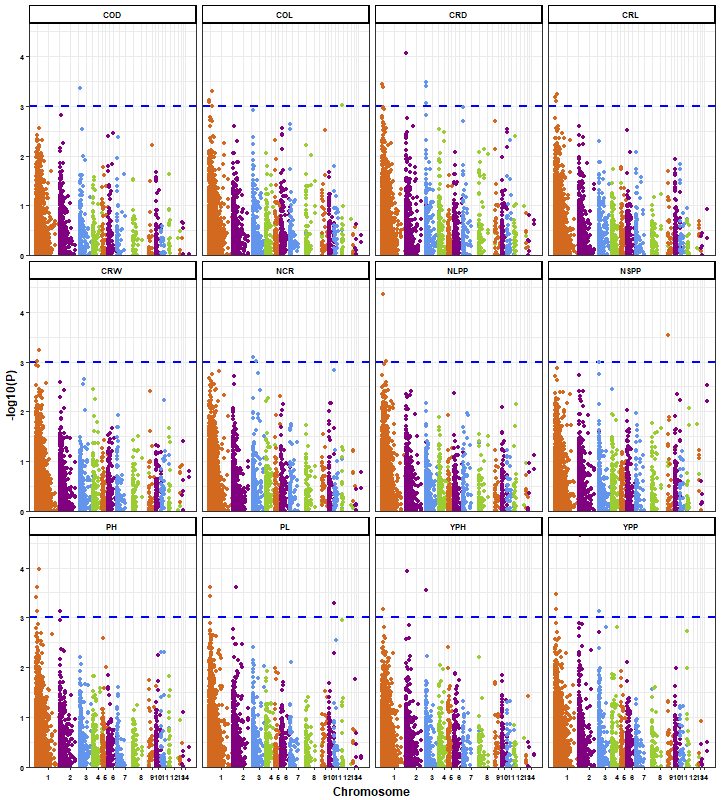


COD= corm diameter (cm), COL= corm length (cm), CRD= cornel diameter 9cm), CRL=cornel length (cm), CRW= cornel weight (g), DM= dry matter, NCR= Number of cormels per plant, PH= plant height (cm), NLPP= number of leaves per plant, NSPP= number of suckers per plant, PL= petiole length (cm), YPH (t/ha)= yield per hectare, and YPP= yield per plants (kg/plant)

**Figure S5-B:** Genome-wide association study across the Nagerian taro landrace collection using 9,442 SNP markers (MFA≥0.01) and **Year 1 (2018) data set**. Manhattan plots showing significant false discovery rate (FDR) adjusted P-value of <0.05 associated with phenotypic traits. The x-axis represents the chromosomes and the y-axis the –log10 (P-values) for marker–trait association. Each point represents the SNP marker. The threshold is set based on the Genetic Type I error calculator (GEC) of the P-values.


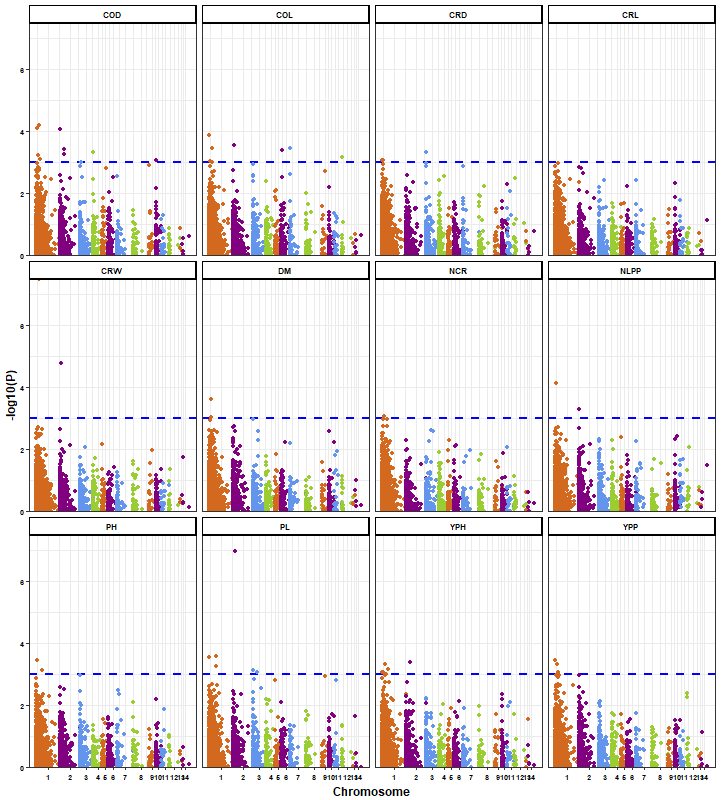


COD= corm diameter (cm), COL= corm length (cm), CRD= cornel diameter 9cm), CRL=cornel length (cm), CRW= cornel weight (g), DM= dry matter, NCR= Number of cormels per plant, PH= plant height (cm), NLPP= number of leaves per plant, NSPP= number of suckers per plant, PL= petiole length (cm), YPH (t/ha)= yield per hectare, and YPP= yield per plants (kg/plant)

**Figure S5-C:** Genome-wide association study across the Nagerian taro landrace collection using 9,442 SNP markers (MFA≥0.01) and **Year 2 (2019) data set**. Manhattan plots showing significant false discovery rate (FDR) adjusted P-value of <0.05 associated with phenotypic traits. The x-axis represents the chromosomes and the y-axis the –log10 (P-values) for marker–trait association. Each point represents the SNP marker. The threshold is set based on the Genetic Type I error calculator (GEC) of the P-values


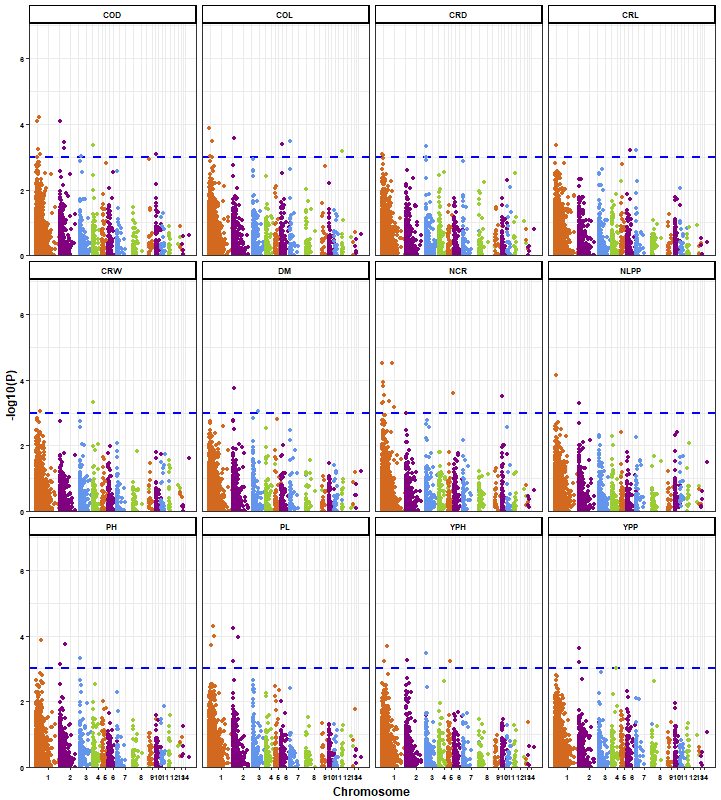


COD= corm diameter (cm), COL= corm length (cm), CRD= cornel diameter 9cm), CRL=cornel length (cm), CRW= cornel weight (g), DM= dry matter, NCR= Number of cormels per plant, PH= plant height (cm), NLPP= number of leaves per plant, NSPP= number of suckers per plant, PL= petiole length (cm), YPH (t/ha)= yield per hectare, and YPP= yield per plants (kg/plant)
